# Decoding Treatment Response and Prognosis in Unresectable Locally Advanced Non–Small Cell Lung Cancer by Integrating Spatiotemporal Heterogeneity From ^18^F-FDG Positron Emission Tomography-Computed Tomography: A Multicenter Study

**DOI:** 10.1200/PO-26-00609

**Published:** 2026-10-02

**Authors:** Yu Ji, Jiaqi Wang, Meixia Zhou, Yaru Wang, Zhengjun Dai, Jingsong Zheng

**Affiliations:** ^1^ Department of Radiology, The Second Qilu Hospital of Shandong University, Jinan, Shandong, China; ^2^ Department of Cancer Medical Center, People's Hospital of Chengwu County, Heze, Shandong, China; ^3^ Department of Radiology, Shandong University, Jinan, Shandong, China; ^4^ Institute of Research and Clinical Innovations, Neusoft Medical Systems Co. Ltd, Beijing, China; ^5^ Center for Nuclear Medicine and Molecular Imaging, Shandong Cancer Hospital and Institute, Shandong First Medical University and Shandong Academy of Medical Sciences, Jinan, China

## Abstract

**Purpose:**

Treatment response in unresectable locally advanced non–small cell lung cancer (LA-NSCLC) is highly variable due to marked intratumoral heterogeneity, while existing predictive biomarkers lack accuracy. We aimed to develop and validate a pretreatment ^18^F-FDG positron emission tomography (PET)-computed tomography (CT)–derived spatiotemporal heterogeneity model to predict treatment response and prognosis in unresectable LA-NSCLC, and to explore its biological underpinnings.

**Materials and Methods:**

In a multicenter cohort of 434 patients with unresectable LA-NSCLC (training: n = 215; internal validation: n = 93; external validation: n = 126), treatment response was classified as responsive or nonresponsive based on initial assessment. Four distinct habitats (PEThigh-CThigh, PETlow-CTlow, PETlow-CThigh, and PEThigh-CTlow) were generated via the Otsu algorithm. Temporal heterogeneity was quantified using metabolic hotspot displacement (normalized distance from the hotspot to the tumor centroid and normalized distance from the hotspot to the tumor periphery) and integrated with spatial radiomics to build a multidimensional spatiotemporal model. Model performance was evaluated against conventional metrics, with interpretability enhanced via nomogram and SHapley Additive exPlanations analysis. Survival outcomes were assessed using Kaplan-Meier curves, and public transcriptomic data were analyzed to characterize underlying pathways.

**Results:**

The spatiotemporal model accurately predicted treatment response with AUCs of 0.822 (training), 0.821 (internal validation), and 0.810 (external validation), significantly outperforming conventional whole-tumor PET-CT models. It maintained robust generalizability across treatment subgroups and effectively stratified patients by progression-free survival and overall survival (*P* < .01). PEThigh-CThigh habitat volume fraction correlated with treatment resistance, whereas PETlow-CTlow habitat correlated with better response. Exploratory pathway analysis highlighted key pathways involving drug metabolism, glycolytic reprogramming, and immune microenvironment remodeling.

**Conclusion:**

This study establishes a noninvasive spatiotemporal heterogeneity biomarker based on ^18^F-FDG PET-CT that reliably predicts treatment response and prognostic outcomes in unresectable LA-NSCLC. It offers valuable insights for personalized clinical decision making and supports precision oncology.

## Graphical abstract

**Figure:**
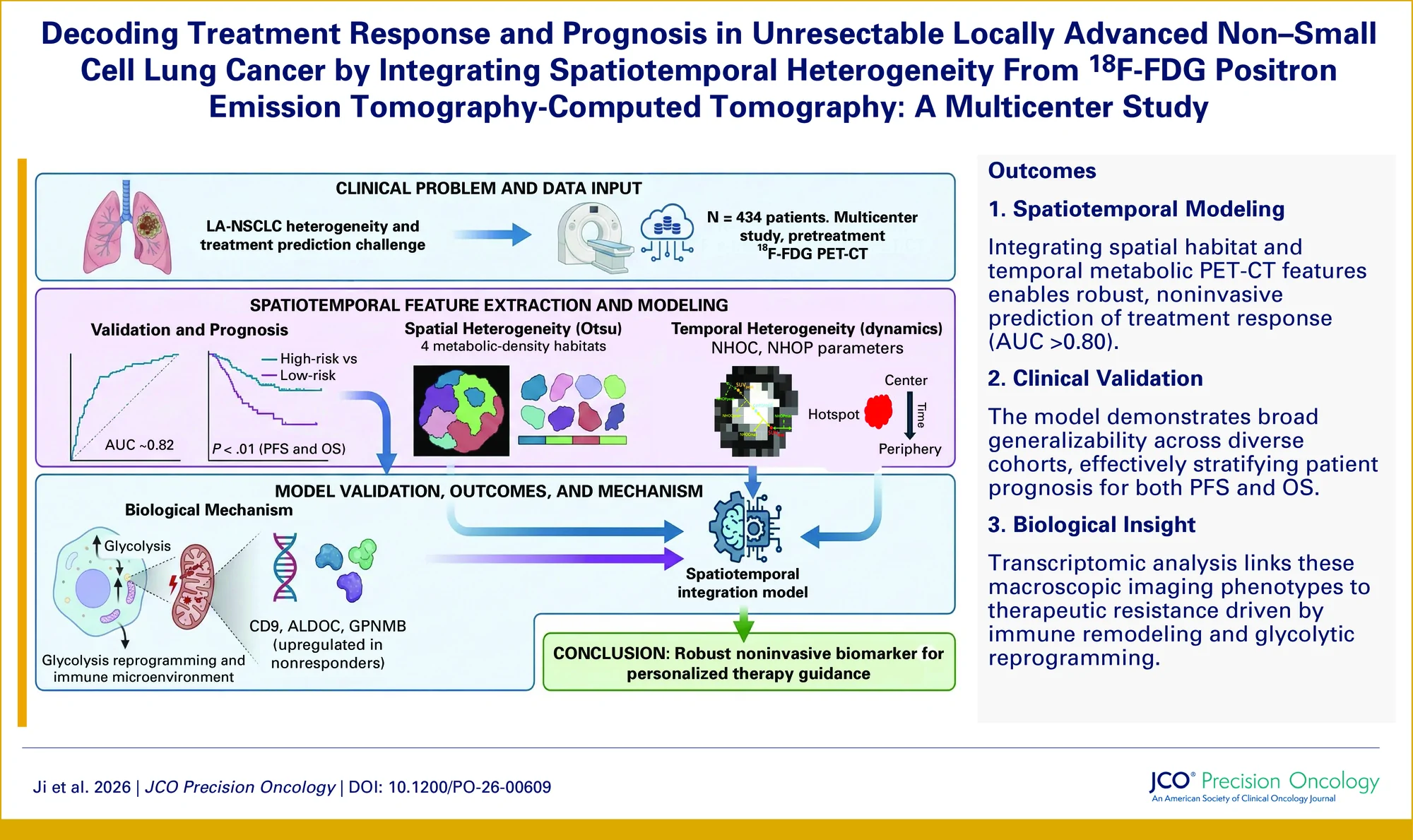

## Introduction

Lung cancer remains a leading cause of cancer-related mortality worldwide.^1,2^ 30% to 40% of patients are still diagnosed at a locally advanced stage, resulting in a 5-year survival rate that remains below 30%.^3,4^ The current standard management for unresectable locally advanced non–small cell lung cancer (LA-NSCLC) involves complex multidisciplinary discussions and intricate treatment methods.^1,2,5^ In this exclusively nonsurgical setting, accurate pretreatment prediction of tumor response is essential to identify patients most likely to benefit, thereby enabling personalized strategies to avoid ineffective interventions, reduce toxicities, and improve survival outcomes.

**Context**

**Key Objective**
Treatment responses to standard nonsurgical therapies in unresectable locally advanced non–small cell lung cancer (LA-NSCLC) are highly heterogeneous. We aimed to develop and validate a multicenter ^18^F-FDG positron emission tomography (PET)-computed tomography (CT)–derived spatiotemporal heterogeneity model to predict treatment response and prognostic risk stratification in this population.
**Knowledge Generated**
By combining spatial habitat radiomics with temporal metabolic hotspot displacement, our spatiotemporal model accurately predicted treatment response across cohorts (AUCs > 0.80), outperforming conventional radiomics models and PET parameters. It significantly stratified patients by progression-free survival and overall survival. PEThigh-CThigh habitats correlated with treatment resistance, with transcriptomic analysis further implicating glycolytic reprogramming and immune microenvironment remodeling.
**Relevance**
This spatiotemporal model provides a noninvasive tool for pretreatment response prediction and risk stratification, enabling personalized therapeutic decision making in unresectable LA-NSCLC.

The assessment of tumor treatment response serves as a cornerstone for evaluating therapeutic efficacy.^6^ However, the limited predictive power of current baseline biomarkers poses a major clinical challenge, primarily attributable to tumor heterogeneity, which drives variable treatment responses through clonal evolutionary selection.^7-9^ ^18^F-FDG PET-CT, as a dual-modality imaging technique that integrates metabolic and anatomic information, provides multidimensional support for the precision medicine of NSCLC.^10,11^ Early studies explored the value of conventional metabolic parameters, such as maximum standardized uptake value (SUVmax), metabolic tumor volume (MTV), and total lesion glycolysis (TLG), along with whole-tumor radiomics for predicting treatment response in NSCLC.^12-15^ However, these approaches yielded contradictory conclusions and failed to adequately capture intratumoral heterogeneity, resulting in limited reliability and interpretability.^12,16^

Habitat analysis addresses the limitations of conventional radiomics techniques by introducing the tumor subregion theory and using unsupervised clustering algorithms to partition lesions into voxel clusters with similar characteristics, thereby enabling the quantification of the intratumoral microenvironment.^16,17^ Furthermore, Jiménez-Sánchez et al^18^ integrated a continuous reaction-diffusion mathematical model with ^18^F-FDG PET imaging to develop a temporal biomarker, NHOC (normalized distance from the hotspot to the tumor centroid), which reveals the evolutionary pattern of metabolic activity shifting from the center toward the periphery over time during tumor growth. Building on this, Hovhannisyan-Baghdasarian et al^19^ further developed NHOP (normalized distance from the hotspot to the tumor periphery), validated the independent prognostic value of both NHOC and NHOP, and demonstrated their robustness and complementary role to radiomic features. Importantly, these parameters are not conventional spatial metrics but biologically grounded surrogates for temporal tumor dynamics. They were derived from a continuous reaction-diffusion mathematical model, and quantify the evolutionary drift of metabolic hotspots from the tumor center toward the periphery during clonal expansion, thereby capturing the temporal dimension of intratumoral heterogeneity from a single static image. However, to our knowledge, no studies have yet been conducted to evaluate the efficacy of unresectable LA-NSCLC using a spatiotemporal heterogeneity model based on ^18^F-FDG PET-CT, and the underlying biological mechanisms linking such heterogeneity to differential treatment responses remain to be elucidated.

Therefore, this study developed a machine learning model based on pretreatment ^18^F-FDG PET-CT images by simultaneously quantifying spatial and temporal heterogeneity in tumors, aiming to predict treatment response in LA-NSCLC and further validate the model's generalizability and prognostic stratification efficacy.

## Materials and Methods

### Study Population

The study protocol was approved by the institutional ethics committee (approval No. KYLL2024487), with informed consent waived due to the retrospective nature. This study retrospectively collected clinical and imaging data from patients with lung cancer treated at two independent medical centers between June 2017 and June 2024. The inclusion criteria were (1) histopathologically confirmed NSCLC (adenocarcinoma or squamous cell carcinoma); (2) clinical stage III disease; (3) availability of complete baseline ^18^F-FDG PET-CT imaging data acquired within 1 week before any antitumor therapy; (4) receipt of standard nonsurgical antitumor treatment; and (5) complete clinical and follow-up data. Exclusion criteria included (1) previous history of antitumor therapy; (2) another primary malignancy; (3) PET-CT image quality insufficient for radiomics analysis; (4) follow-up CT image quality inadequate for response assessment; and (5) presence of a large intratumoral cavity exceeding 2.0 cm in diameter. The TNM staging of LA-NSCLC in this study was based on the International Association for the Study of Lung Cancer Lung Cancer Staging Project.^20^ Patients were stratified into two treatment groups: (1) a definitive chemoradiotherapy group, who received concurrent or sequential platinum-based chemotherapy combined with thoracic radiotherapy as curative-intent treatment; and (2) a nonchemoradiotherapy group, who received heterogeneous nonsurgical regimens, including systemic chemotherapy alone, immunotherapy, targeted therapy, or individualized protocols due to medical inoperability. All patients were deemed unresectable or medically unfit for surgery after multidisciplinary tumor board assessment. The detailed treatment distribution and patient stratification are described in the Supplementary Materials. The specific flowchart and design pathway of the study are presented in Figures 1 and 2, respectively.

**Fig 1.**
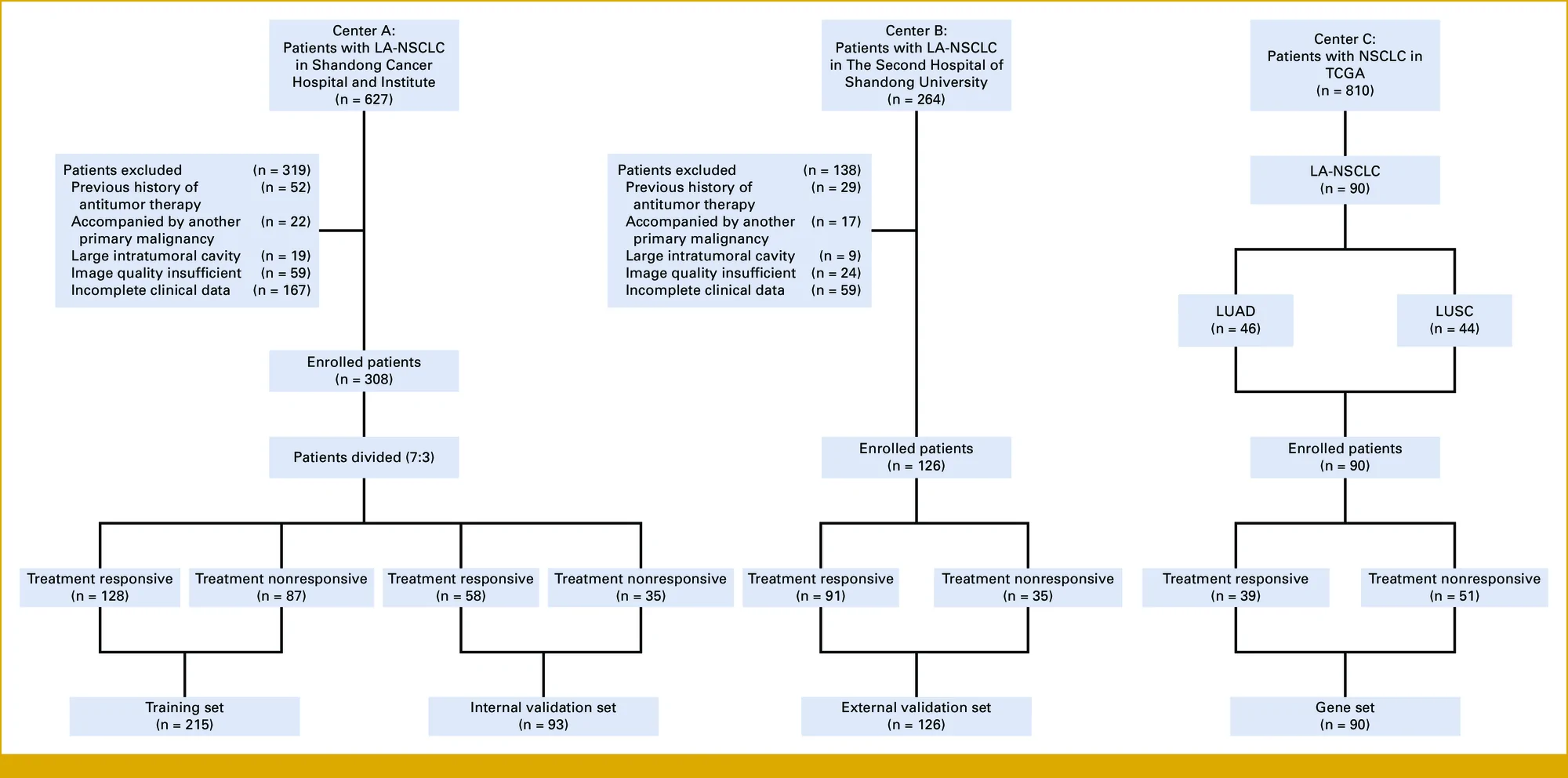
Flowchart of patient enrollment and study profile. LA-NSCLC, locally advanced non–small cell lung cancer; TCGA, The Cancer Genome Atlas.

**Fig 2.**
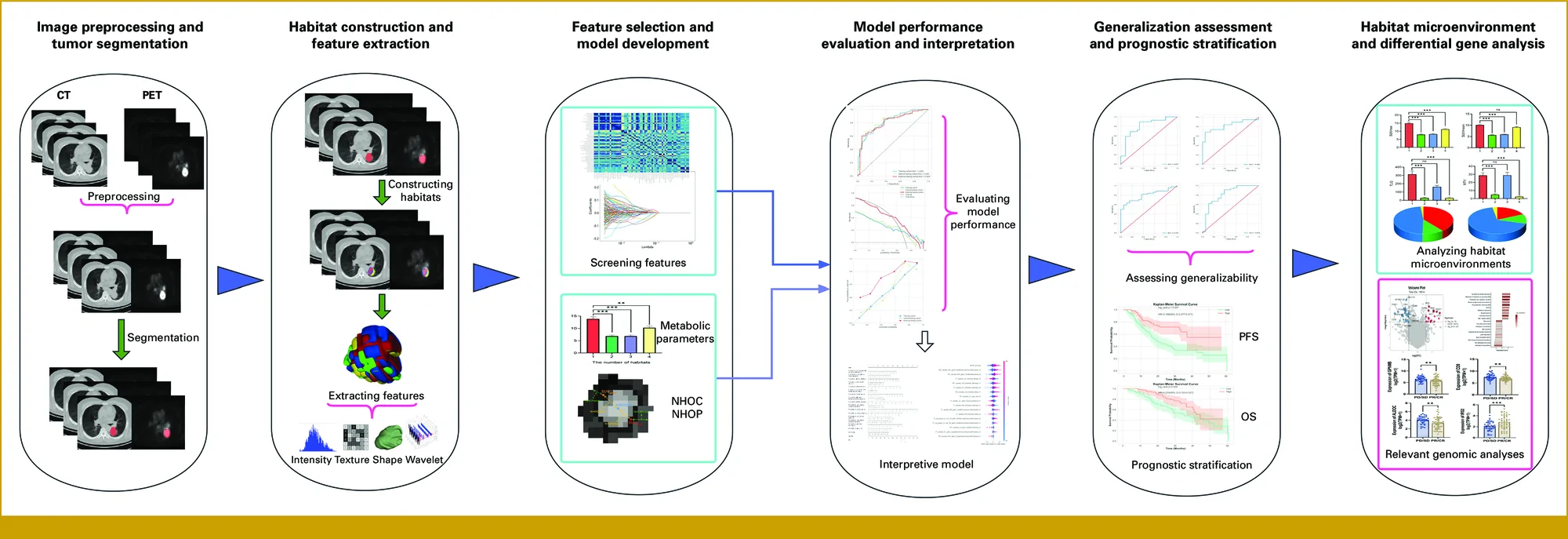
Study design and workflow. The workflow began with the acquisition, preprocessing, and segmentation of baseline ^18^F-FDG PET-CT images from patients with LA-NSCLC. Subsequently, the Otsu algorithm was applied to generate four distinct habitats, from which spatial radiomic features were extracted. Following this, temporal heterogeneity was quantified using two parameters (NHOC and NHOP), after which feature selection was performed. A multidimensional heterogeneity prediction model was then developed and evaluated. Nomograms and SHAP analysis were used to interpret and visualize the model's decision rules. The model's generalizability and prognostic stratification capability were further assessed through multisubgroup validation. Finally, microenvironmental differences among the habitats were analyzed alongside differentially expressed genes identified from TCGA to explore potential biological mechanisms underlying treatment response. LA-NSCLC, locally advanced non–small cell lung cancer; NHOC, normalized distance from the hotspot to the tumor centroid; NHOP, normalized distance from the hotspot to the tumor periphery; PET-CT, positron emission tomography-computed tomography; SHAP, SHapley Additive exPlanations; TCGA, The Cancer Genome Atlas.

### Treatment Response Assessment and Follow-Up

Early treatment response was assessed by contrast-enhanced CT at 6-10 weeks after therapy. The definitive chemoradiotherapy group was evaluated per RECIST 1.1^21^; the nonchemoradiotherapy group was evaluated by iRECIST (if receiving immunotherapy) or RECIST 1.1. Complete response/immune complete response and partial response/immune partial response were classified as responsive, and stable disease/immune stable disease and progressive disease/immune confirmed progressive disease as nonresponsive. The treatment response assessment workflow is shown in the Data Supplement (Fig S1). Progression-free survival (PFS) and overall survival (OS) were defined as the time from diagnosis to disease progression or death, and to death or last follow-up, respectively. Follow-up protocols are provided in the Supplementary Materials.

### ^18^F-FDG PET-CT Image Acquisition, Preprocessing, and Segmentation

All scans were performed within 1 week before therapy. Detailed acquisition parameters, preprocessing, and segmentation protocols are provided in the Supplementary Materials. Tumor segmentation and all subsequent radiomic analyses were restricted to the primary pulmonary tumor, as the study aimed to evaluate intrinsic primary tumor heterogeneity as a predictor of systemic treatment response.

### Tumor Habitat Generation

The habitats in this study were constructed using the Otsu threshold segmentation method. For the PET images, the Otsu algorithm calculated the threshold that maximizes the interclass variance of the SUV value distribution within the volume of interest (VOI). This divides the VOI into high-metabolism and low-metabolism subregions. The same procedure was applied to the CT images to partition the VOI into high-density and low-density subregions based on Hounsfield unit values. Finally, the PET-derived subregions and CT-derived density subregions were intersected to generate four distinct habitats: PEThigh-CThigh, PETlow-CTlow, PETlow-CThigh, and PEThigh-CTlow.

### Radiomics Feature Extraction and Selection

Radiomic feature extraction was performed using the integrated analysis pipeline within the PyRadiomics software package,^22^ strictly adhering to the guidelines of the Image Biomarker Standardization Initiative to ensure feature comparability and reproducibility. Features were extracted from four habitat subregions and the entire tumor volume across both PET and CT modalities. Detailed methodologies for radiomics feature extraction and selection are provided in the Supplementary Materials.

### Calculation of Time-Heterogeneous Parameters NHOC and NHOP

The standardized hotspot-to-centroid distance (NHOC) was defined as the three-dimensional Euclidean distance from the point of maximum metabolic activity within the tumor to its geometric centroid, normalized by the radius of an equivalent sphere of identical volume. Similarly, the standardized hotspot-to-periphery distance (NHOP) was defined as the shortest distance from the metabolic hotspot to the nearest tumor boundary, also normalized by the equivalent sphere radius.^19^ This normalization yields dimensionless quantities bounded between 0 and 1, ensuring that both metrics reflect the relative spatial positioning of the metabolic hotspot within the tumor architecture rather than absolute tumor dimensions. To exclude potential confounding by tumor volume, the correlation between NHOP-SUVmax and MTV was assessed, and the variance inflation factor was calculated in the multivariate logistic regression (LR) model. The specific calculation methods, formulas, and collinearity analysis results are provided in the Supplementary Materials.

### Model Development

This study constructed four distinct predictive models for treatment response based on multimodal radiomic features and clinical characteristics: (1) spatiotemporal heterogeneity model; (2) habitat model; (3) traditional whole-tumor radiomics model; and (4) clinical/metabolic model. Model performance was evaluated via ROC curves and calibration curves, with clinical utility assessed by using decision curve analysis (DCA). After the overall performance evaluation, subgroup analyses based on pathologic subtype and treatment modality were performed to assess the model's generalizability. Using the median score of the spatiotemporal heterogeneity joint model as the cutoff, patients were stratified into high-risk and low-risk groups. Kaplan-Meier survival analysis and the log-rank test were then applied in the training, internal validation, and external validation sets to analyze survival differences between the groups, evaluating the model's prognostic stratification capability.

### Treatment Response–Related Gene Analysis

Transcriptomic and corresponding clinical data from patients with lung adenocarcinoma (LUAD) and lung squamous cell carcinoma (lung squamous cell carcinoma [LUSC]; n = 810) were obtained from The Cancer Genome Atlas (TCGA) database.^23^ From this cohort, we identified a subset of patients with LA-NSCLC (n = 90), who were further categorized into a treatment responsive group (n = 39) and a nonresponsive group (n = 51) based on their treatment outcomes. It is crucial to emphasize that the primary purpose of using TCGA data in this study was not to serve as an external validation set, but rather as an exploratory tool to identify potential genes and pathways associated with the treatment response phenotype from a public genomics resource, thereby generating mechanistic hypotheses for the heterogeneity captured by our imaging features. Detailed methodologies for gene analysis are provided in Supplementary Materials.

### Statistical Analysis

Statistical analyses in this study were performed using R language (version 3.5.3)^24^ in accordance with the International Committee of Medical Journal Editors guidelines for statistical reporting. Detailed methodologies are provided in the Supplementary Materials.

### Ethics Statement

This study was performed in accordance with the Declaration of Helsinki. Approval was granted by the Ethics Committees of The Second Qilu Hospital of Shandong University (approval No. KYLL2024487).

### Consent Statement

Because of the retrospective nature of the study, the requirement for informed consent was waived by the ethics committee. This study did not include animal experiments. Consent for publication is not applicable because no individually identifiable patient data are presented.

## Results

### Baseline Characteristics

Baseline characteristics were compared across groups. Intergroup analysis revealed significant differences in lung lobe involvement, ProGRP, TLG, and MTV (all *P* < .05), while remaining features were balanced (*P* > .05; Table 1). Univariate analysis indicated that MTV, NHOP-SUVmax, and NHOP-SUVpeak showed significant correlations with treatment response. Multivariate LR further confirmed NHOP-SUVmax as the sole independent predictor of treatment response (OR = 14.058 [95% CI, 3.611 to 54.733]; *P* < .001; Data Supplement, Table S1).

**Table 1.** Clinical and Metabolic Characteristics of Study Sets

Variable	Train Set (n = 215)	Internal Validation Set (n = 93)	External Validation Set (n = 126)	*P*
Age, years	63.9 ± 8.6	63.8 ± 9.2	61.3 ± 9.3	.360
Sex				.423
Male	184 (85.6%)	77 (82.8%)	101 (80.2%)	
Female	31 (14.4%)	16 (17.2%)	25 (19.8%)	
T stage				.312
I	26 (12.1%)	10 (10.8%)	14 (11.1%)	
II	66 (30.7%)	32 (34.4%)	53 (42.1%)	
III	59 (27.4%)	20 (21.5%)	32 (25.4%)	
IV	64 (29.8%)	31 (33.3%)	27 (21.4%)	
N stage				.158
0	11 (5.1%)	3 (3.2%)	11 (8.7%)	
I	26 (12.1%)	7 (7.5%)	16 (12.7%)	
II	102 (47.4%)	47 (50.5%)	68 (54.0%)	
III	76 (35.3%)	36 (38.7%)	31 (24.6%)	
Clinical stage				.090
I	89 (41.4%)	33 (35.5%)	72 (57.1%)	
II	89 (41.4%)	42 (45.2%)	43 (34.1%)	
III	37 (17.2%)	18 (19.4%)	11 (8.7%)	
Smoking				.708
Yes	60 (27.9%)	30 (32.3%)	35 (27.8%)	
No	155 (72.1%)	63 (67.7%)	91 (72.2%)	
Pathology				.054
LUAD	86 (40.0%)	31 (33.3%)	62 (49.2%)	
LUSC	129 (60.0%)	62 (66.7%)	64 (50.8%)	
Tumor location				.899
Central	135 (62.8%)	58 (62.4%)	76 (60.3%)	
Peripheral	80 (37.2%)	35 (37.6%)	50 (39.7%)	
Lung lobe involvement				.020
LUL	54 (25.1%)	27 (29.0%)	35 (27.8%)	
LLL	34 (15.8%)	15 (16.1%)	31 (24.6%)	
RUL	68 (31.6%)	28 (30.1%)	21 (16.7%)	
RML	11 (5.1%)	5 (5.4%)	11 (8.7%)	
RLL	48 (22.3%)	16 (17.2%)	28 (22.2%)	
CEA				.397
Normal (≤5.0 μg/L)	103 (47.9%)	37 (39.8%)	55 (43.7%)	
Abnormal (>5.0 μg/L)	112 (52.1%)	56 (60.2%)	71 (56.3%)	
NSE				.654
Normal (≤16.3 ng/mL)	137 (63.7%)	57 (61.3%)	74 (58.7%)	
Abnormal (>16.3 ng/mL)	78 (36.3%)	36 (38.7%)	52 (41.3%)	
CYFRA21-1				.281
Normal (≤3.3 ng/mL)	80 (37.2%)	28 (30.1%)	51 (40.5%)	
Abnormal (>3.3 ng/mL)	135 (62.8%)	65 (69.9%)	75 (59.5%)	
ProGRP				.005
Normal (≤69.2 pg/mL)	180 (83.7%)	68 (73.1%)	113 (89.7%)	
Abnormal (>69.2 pg/mL)	35 (16.3%)	25 (26.9%)	13 (10.3%)	
SCCA				.313
Normal (≤2.7 ng/mL)	179 (83.3%)	77 (82.8%)	112 (88.9%)	
Abnormal (>2.7 ng/mL)	36 (16.7%)	16 (17.2%)	14 (11.1%)	
Treatment method				.704
Definitive chemoradiotherapy	118 (54.9%)	53 (57.0%)	63 (50.0%)	
Nonchemoradiotherapy	97 (45.1%)	40 (43.0%)	63 (50.0%)	
Therapeutic effect				
Response	128 (59.5%)	58 (62.4%)	91 (72.2%)	.206
Nonresponse	87 (40.5%)	35 (37.6%)	35 (27.8%)	
SUVmax	15.08 ± 0.41	15.23 ± 0.63	14.28 ± 0.59	.144
SUVpeak	12.87 ± 0.36	13.20 ± 0.57	12.28 ± 0.48	.166
SUVmean	7.72 ± 0.21	7.90 ± 0.30	7.95 ± 0.29	.697
TLG, g	533.83 ± 43.07	504.05 ± 71.96	381.12 ± 52.05	.007
MTV, cm^3^	67.97 ± 5.44	60.63 ± 8.14	42.97 ± 5.26	.002
NHOC-SUVmax	0.65 (0.46-0.80)	0.60 (0.50-0.80)	0.64 (0.41-0.84)	.979
NHOC-SUVpeak	0.55 (0.35-0.69)	0.60 (0.38-0.74)	0.52 (0.31-0.73)	.536
NHOP-SUVmax	0.32 (0.22-0.46)	0.34 (0.22-0.45)	0.32 (0.21-0.44)	.928
NHOP-SUVpeak	0.42 (0.33-0.55)	0.40 (0.32-0.50)	0.41 (0.31-0.54)	.774

NOTE. Unless otherwise specified, data are numbers of participants, with percentages in parentheses.

Abbreviations: CEA, carcinoembryonic antigen; CYFRA21-1, soluble fragment of cytokeratin 19; LLL, left lower lobe; LUAD, lung adenocarcinoma; LUL, left upper lobe; LUSC, lung squamous carcinoma; MTV, metabolic tumor volume; NHOC, normalized distance from the hotspot to the tumor centroid; NHOP, normalized distance from the hotspot to the tumor periphery; NSE, neuron-specific enolase; ProGRP, pro-gastrin-releasing peptide; RLL, right lower lobe; RML, right middle lobe; RUL, right upper lobe; SCCA, squamous cell carcinoma antigen; SUV, standardized uptake value; TLG, total lesion glycolysis.

### Performance Evaluation of Predictive Models

A total of 16 radiomic features with nonzero coefficients were selected from the habitats for model construction (Data Supplement, Table S2). Among six evaluated machine learning algorithms, LR demonstrated optimal and stable performance and was thus chosen as the final classifier. The habitat model based on LR achieved AUCs of 0.803 (training), 0.781 (internal validation), and 0.781 (external validation; Data Supplement, Figs S2-S4). The detailed results are available in the Data Supplement (Table S3).

The habitat model achieved superior efficacy over both the whole-tumor radiomics and clinical/metabolic models (Fig 3, Data Supplement, Figs S5, S6, and Table S3). Furthermore, the multidimensional spatiotemporal heterogeneity model, which integrated spatial (habitat radiomics) and temporal (NHOC/NHOP) heterogeneity features, exhibited enhanced predictive performance, yielding AUCs of 0.822 (95% CI, 0.778 to 0.862), 0.821 (95% CI, 0.746 to 0.892), and 0.810 (95% CI, 0.740 to 0.874) across the three cohorts. This model showed excellent calibration and the highest net clinical benefit in DCA (Fig 3). A nomogram and SHapley Additive exPlanations (SHAP) analysis were constructed to enhance model interpretability and clinical translation (Data Supplement, Fig S7). Representative patient cases are presented to illustrate the clinical application value and translational potential of the spatiotemporal heterogeneity model in individualized treatment response prediction (Data Supplement, Fig S8).

**Fig 3.**
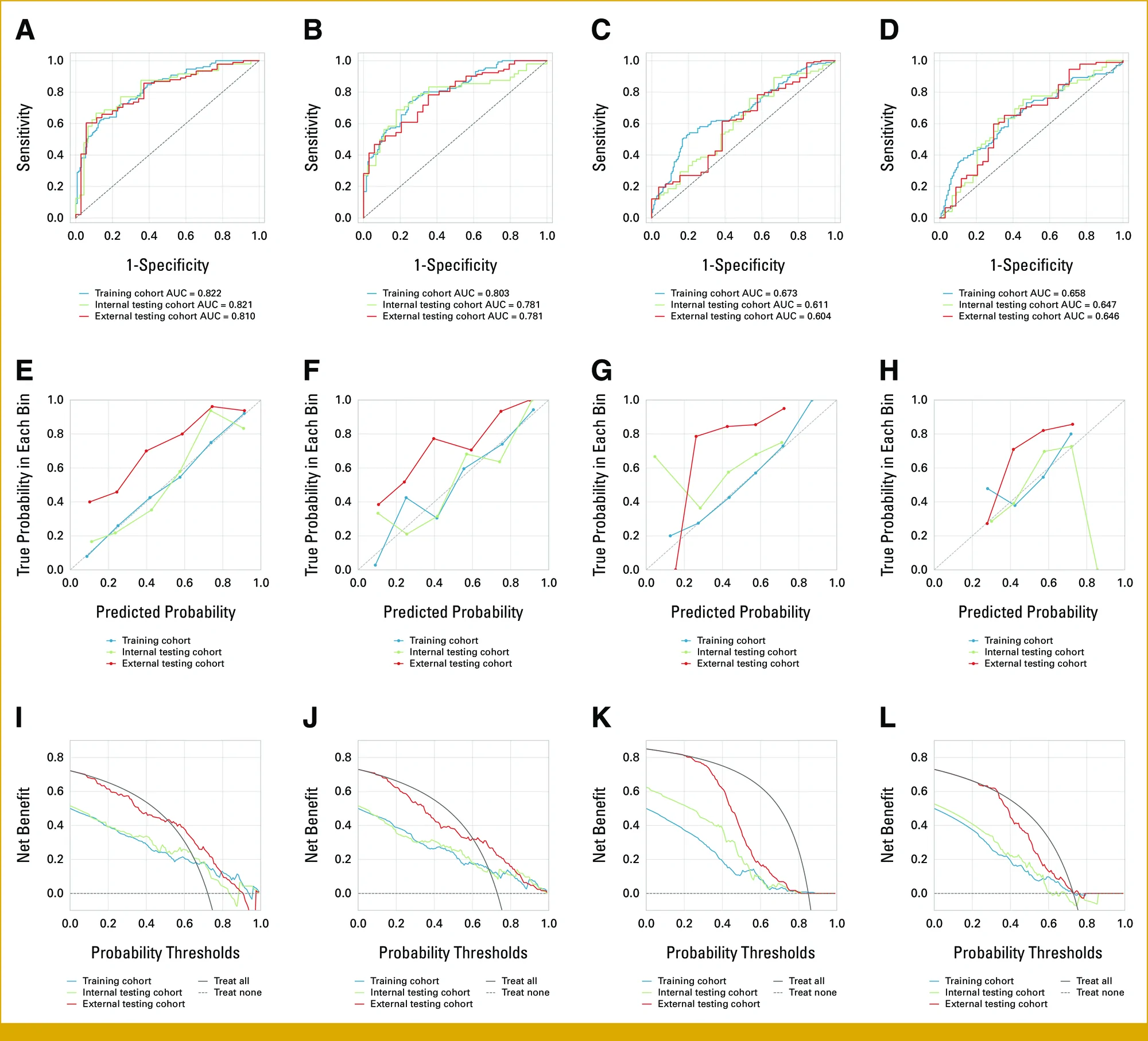
The performance of the spatiotemporal heterogeneity model (A, E, I), habitat model (B, F, J), whole-tumor radiomics model (C, G, K), and clinical/metabolic model (D, H, L) was comprehensively compared. The spatiotemporal heterogeneity model demonstrated superior predictive performance, as evidenced by its receiver operating characteristic (A-D), calibration (E-H), and decision analysis (I-L) curves. Notably, within a reasonable threshold probability range, the spatiotemporal heterogeneity model consistently provided a greater net benefit than the other models.

The spatiotemporal heterogeneity model was validated across subgroups defined by histology (LUSC *v* LUAD) and treatment approach (definitive chemoradiotherapy *v* nonchemoradiotherapy), demonstrating robust and consistent efficacy (maximum intersubgroup ΔAUC < 0.06; Data Supplement, Fig S9 and Table S4). After adjusting for histology and treatment modality, spatiotemporal features remained independent predictors (Data Supplement, Table S1), with no significant interactions (P for interaction > .05). These findings indicate consistent predictive performance across diverse pathologic subtypes and therapeutic regimens.

### Analysis of Metabolic and Spatial Characteristics Across Habitats

Four distinct habitats were successfully constructed, each corresponding to a unique combination of metabolic activity and anatomic density. Metabolic level analysis confirmed excellent consistency across all cohorts. Representative features and detailed statistical comparisons of the four habitats are illustrated in the Data Supplement (Fig S10 and Table S5). Spatial quantification of the habitats, measured in units of 1,000 voxels, demonstrated a significant negative correlation between the volume proportion of Habitat 1 and treatment response (OR = 0.063 [95% CI, 0.008 to 0.483]; *P* < .01). Conversely, the volume proportion of Habitat 2 showed a positive correlation with treatment response (OR = 9.668 [95% CI, 1.073 to 87.241]; *P* < .05). However, the other habitats were not associated with therapeutic outcomes (Data Supplement, Table S6).

### Prognosis Analysis

The median follow-up times for PFS and OS were 19.2 months (range, 1.4-68.1 months) and 26.7 months (range, 2.7-68.1 months), respectively. Patients were stratified into high-risk and low-risk groups based on the median risk score derived from the spatiotemporal heterogeneity joint model. Survival analysis demonstrated statistically significant differences in PFS and OS between the two groups across all data sets (training set, *P* < .001; internal validation, *P* < .001; external validation, *P* < .01; Fig 4).

**FIG 4.**
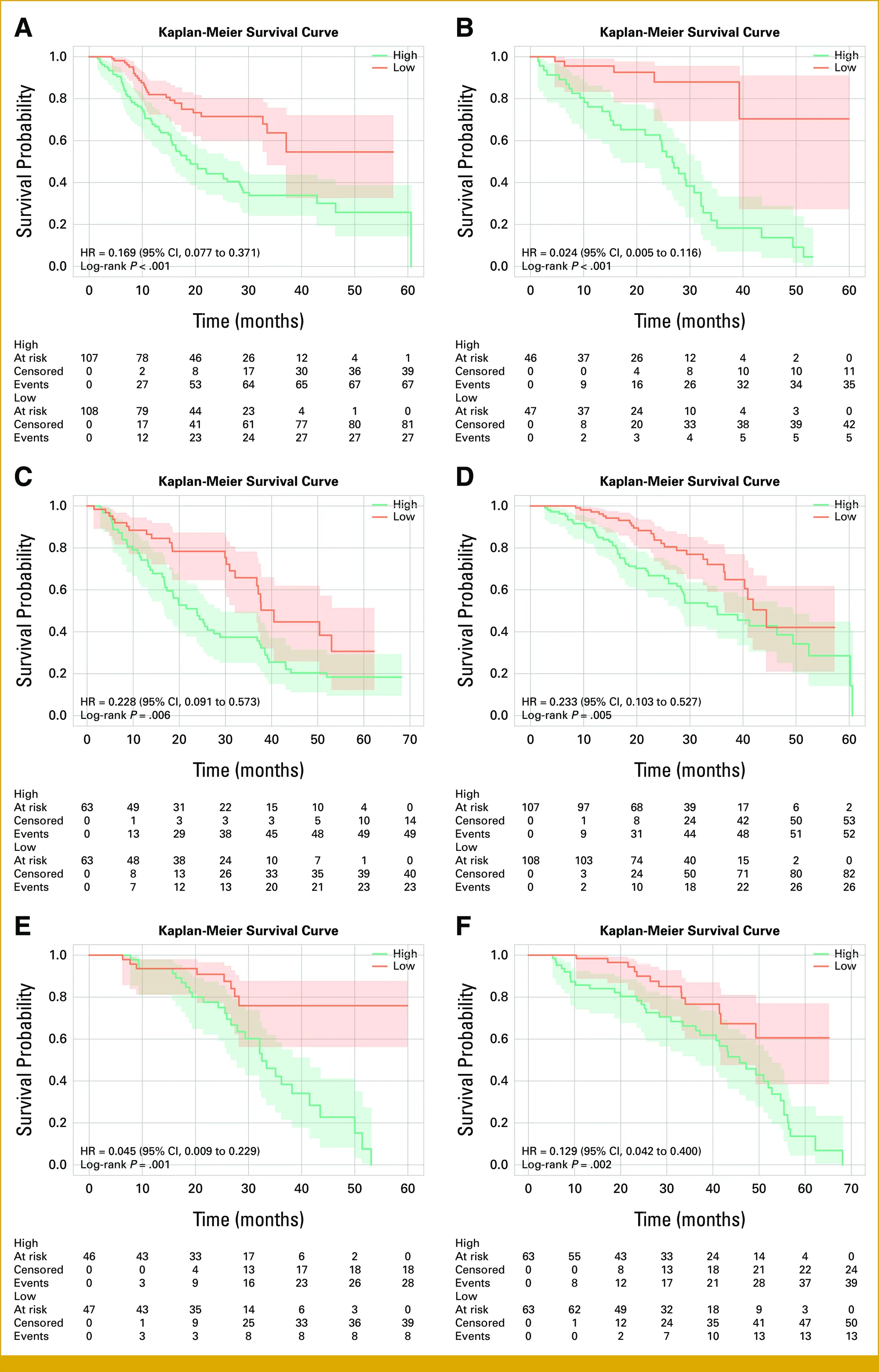
Kaplan-Meier curves depict PFS (A-C) and OS (D-F) in the training set (A, D), internal validation set (B, E), and external validation set (C, F). Survival outcomes were stratified using risk scores derived from the spatiotemporal heterogeneity model, which integrated features from spatial habitat radiomics and temporal heterogeneity parameters (NHOC and NHOP). Patients were divided into low-risk (red curve) and high-risk (green curve) groups based on the median value of the model-derived scores. NHOC, normalized distance from the hotspot to the tumor centroid; NHOP, normalized distance from the hotspot to the tumor periphery; OS, overall survival; PFS, progression-free survival.

### Differentially Expressed Gene Analysis

Integration of transcriptomic data from LUAD and LUSC subtypes followed by differential expression analysis identified 42 differentially expressed genes. Intersection with a glycolytic gene set revealed four glycolysis-related genes (*GPNMB*, *CD9*, *ALDOC*, and *IRS2*). Pathway enrichment analysis indicated significant involvement in drug metabolism, pentose and glucuronate interconversions, ascorbate and aldarate metabolism, and glutathione metabolism (Fig 5).

**Fig 5.**
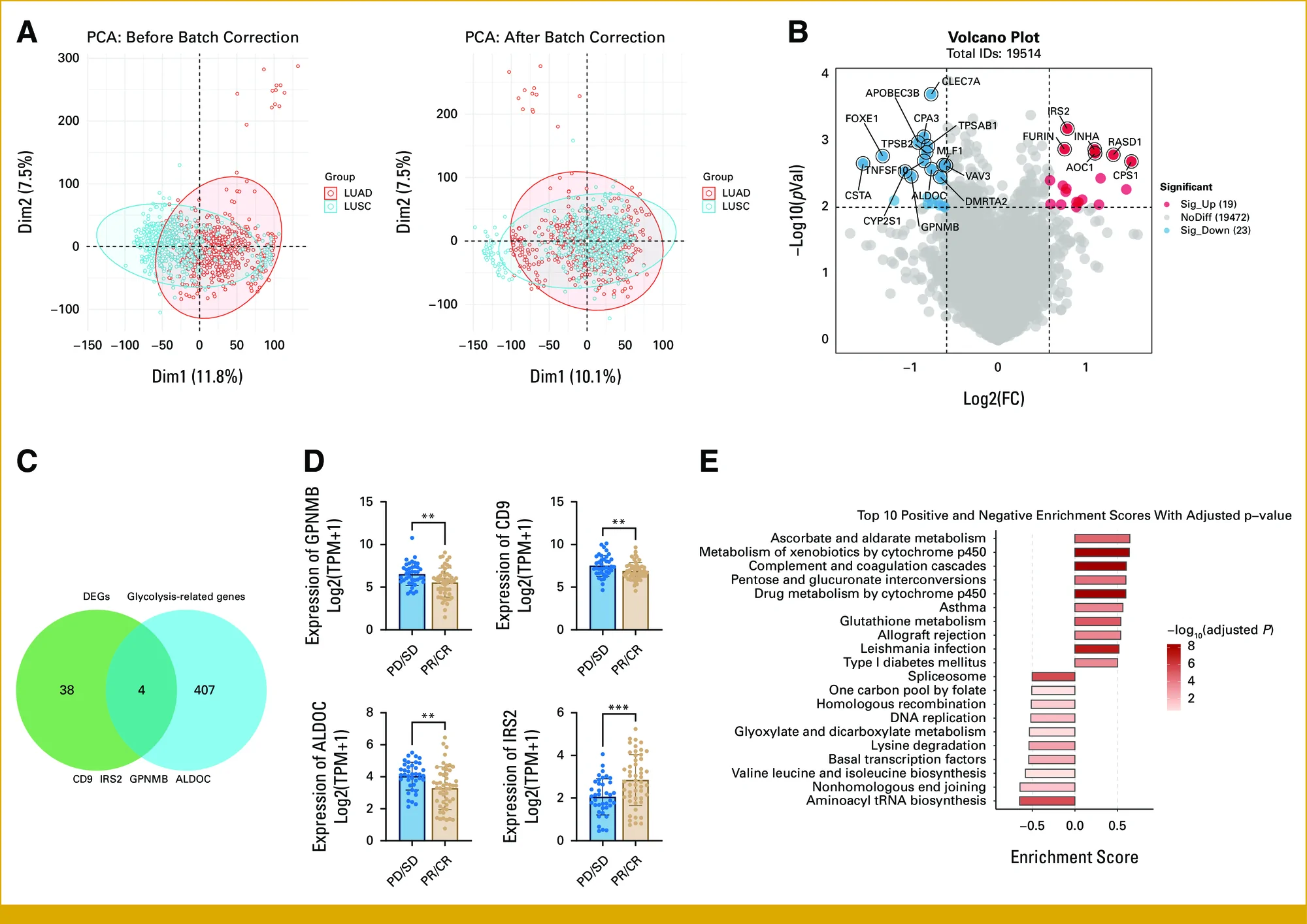
Correlation analysis of differentially expressed genes associated with treatment response in LA-NSCLC. (A) PCA illustrates the distribution of LUAD and LUSC samples before and after batch-effect correction using TCGA data. (B) Volcano plot displays the differentially expressed genes. (C) A Venn diagram demonstrates the identification of four glycolysis-related genes (*GPNMB*, *CD9*, *ALDOC*, and *IRS2*) associated with differential treatment response in LA-NSCLC, obtained from the intersection between differentially expressed genes and a glycolysis-related gene set. (D) Among these, *GPNMB*, *CD9*, and *ALDOC* were upregulated in the treatment nonresponsive group, whereas *IRS2* was upregulated in the treatment responsive group. (E) Pathway enrichment analysis revealed that the differentially expressed genes were significantly enriched in important metabolic pathways, including drug metabolism, pentose and glucuronate interconversions, ascorbate and aldarate metabolism, and glutathione metabolism. *P* < .05 was considered statistically significant. ***P < .001, **P < .01, *P < .05. CR, complete response; LA-NSCLC, locally advanced non–small cell lung cancer; LUAD, lung adenocarcinoma; LUSC, lung squamous cell carcinoma; PCA, principal component analysis; PD, progressive disease; PR, partial response; SD, stable disease; TCGA, The Cancer Genome Atlas.

## Discussion

This study developed a novel predictive model based on pretreatment baseline ^18^F-FDG PET-CT images from a multicenter cohort, leveraging intratumoral multidimensional spatiotemporal heterogeneity to achieve noninvasive assessment of treatment response in unresectable LA-NSCLC. The spatiotemporal model consistently outperformed single-scale models across all three cohorts. The model showed consistent performance across subgroups defined by histopathology and treatment modalities, attesting to its generalizability. Moreover, the model exhibited significant prognostic stratification power, with the high-risk group showing markedly inferior PFS and OS compared with the low-risk group, indicating its dual utility in predicting treatment response and guiding individualized prognosis.

Tumor spatiotemporal heterogeneity encompasses both spatial heterogeneity and temporal heterogeneity, both of which influence treatment response.^25^ Conventional radiomics or habitat analysis has predominantly focused on spatial dimensions, inevitably overlooking the dynamically evolving nature of tumors.^26^ Studies have confirmed that as tumors progress, highly proliferative and invasive clones migrate toward the periphery, resulting in a shift of the FDG uptake hotspot from the tumor center toward the edge.^18,19^ As temporal-dimensional features independent of radiomics, NHOC and NHOP demonstrate high robustness and significant clinical applicability, a conclusion supported by our findings. Furthermore, our study identified that NHOP exhibited a stronger correlation with treatment response, particularly NHOP-SUVmax, which emerged as the sole independent predictor of treatment efficacy, consistent with reports by Jiménez-Londoño et al.^27^ Although NHOP-SUVpeak was significant in univariate analysis, it lost significance in the multivariate model, indicating that its predictive information was largely redundant with NHOP-SUVmax. Biologically, treatment resistance in LA-NSCLC is frequently driven by focal, highly aggressive subclones; the single-voxel maximum metric (SUVmax) captures their microenvironmental displacement more sensitively than the regionally averaged SUVpeak, which tends to dilute fine-grained spatial shifts.

After adjusting for covariates and their interactions, the model continued to demonstrate robust predictive performance across distinct subgroups. Stratifying patients by the model score significantly distinguished PFS and OS, achieving precise prognostic stratification. This capability likely stems from the model's effective quantification of intratumoral heterogeneity and its successful classification of treatment response, a surrogate end point for long-term survival. To facilitate clinical translation, the nomogram and SHAP analysis simplify model implementation and enhance interpretability. However, the model is intended as a complementary predictive biomarker and decision-support adjunct, not a standalone clinical decision algorithm. Its outputs should be interpreted by experienced thoracic oncologists and integrated into holistic clinical assessment alongside performance status, molecular profiling, and comorbidities.

To elucidate the biological mechanisms underlying the model's predictive and prognostic capabilities, it is essential to clarify that its value resides in the relative spatial distribution and temporal evolutionary patterns of the habitats, rather than the isolated presence of high-FDG regions. Indeed, multivariate analysis confirmed that conventional metabolic parameters lacked independent predictive significance. Instead, by integrating multiregional radiomic features across all four habitats and quantifying the spatial displacement of metabolic hotspots, the model captures the intrinsic spatial complexity and evolutionary dynamics of the tumor microenvironment. Furthermore, the inverse prognostic relationship observed between the PEThigh-CThigh and PETlow-CTlow volume fractions underscores that therapeutic outcomes are dictated by the dynamic balance among coexisting microenvironments, rather than the absolute metabolic activity of any single subregion.

At the habitat level, the volume proportion of PEThigh-CThigh was positively correlated with treatment resistance, whereas PETlow-CTlow correlated with better response. Notably, the volume fraction of Habitat 1 was inversely associated with treatment response, whereas the absolute voxel count was not. This discrepancy reflects the fact that volume fraction captures the relative dominance of a microenvironment within the tumor ecosystem, whereas voxel count merely reflects absolute abundance and is confounded by total tumor volume. Treatment response is governed by the balance of coexisting habitats, not by the absolute volume of any single subregion. We hypothesize that high-metabolism habitats represent the hyperproliferative tumor core linked to accelerated glucose metabolism, hypoxia, and angiogenesis, exhibiting enhanced DNA repair and drug resistance. Conversely, low-metabolism subregions may correspond to immune-infiltrated or necrotic areas with synergistic treatment sensitivity.

At the molecular pathway level, four glycolysis-related genes were identified: *GPNMB*, *CD9*, and *ALDOC* were upregulated in the nonresponsive group. These genes are known to enhance the Warburg effect, accelerating glycolysis to initiate tumor drug resistance, consistent with existing literature.^28,29^ By contrast, *IRS2* was upregulated in the responsive group. Although *IRS2* participates in the PI3K/Akt pathway, its synergistic relationship with EGFR expression may explain its upregulation in responsive patients, given the efficacy of TKIs in EGFR-mutant LA-NSCLC.^30^ Furthermore, pathway enrichment analysis revealed significant involvement in drug metabolism, pentose and glucuronate interconversions, glutathione metabolism, and ascorbate and aldarate metabolism pathways. These results highlight the important roles of metabolic heterogeneity, glycolytic reprogramming, and immune microenvironment remodeling in driving treatment resistance and immune escape in LA-NSCLC. It is important to recognize that these biological interpretations represent an evidence-based framework derived from our model, not an exhaustive explanation of NSCLC tumor biology. Factors such as circulating tumor DNA dynamics, tumor microbiome, spatial transcriptomics, and stromal-immune interactions lie beyond the resolution of conventional PET-CT and were not captured in this study. Furthermore, our genomic correlations relied on population-level TCGA data rather than patient-specific radiogenomic validation. Thus, although the biological plausibility of our model has been sufficiently supported, the full spectrum of biological determinants governing treatment response remains to be elucidated.

This study has several limitations that should be acknowledged. First, the retrospective design introduces potential selection bias and precludes systematic molecular profiling, as genomic data were not systematically collected across the entire cohort. Prospective studies with larger, well-stratified cohorts are required to confirm the accuracy of the spatiotemporal heterogeneity model while evaluating the influence of specific mutational profiles on its predictive performance. Second, the exclusive enrollment of patients with unresectable LA-NSCLC limits generalizability to the resectable subset. Future studies should therefore validate the model in neoadjuvant-treated, surgically resected cohorts to determine its applicability across the full Stage III spectrum. Third, the genomic correlation analysis relied on population-level TCGA associations rather than patient-specific radiogenomic validation, precluding direct imaging-to-biopsy correlations; nevertheless, this exploratory analysis generated biologically plausible hypotheses regarding glycolytic mechanisms warranting paired multiomics validation in future prospective studies. Fourth, although the nomogram and SHAP enhance interpretability, the model currently requires specialized radiomics software and machine learning expertise, limiting immediate clinical deployment. Automated, clinician-friendly pipelines are needed to facilitate routine use. Finally, our spatiotemporal heterogeneity model was derived exclusively from the primary tumor, and the potential prognostic value of heterogeneity differences between the primary tumor and involved regional lymph nodes remains unexplored. Future studies should evaluate combined primary-nodal radiomic models to determine whether these signatures can discriminate between local-regional control and distant dissemination, thereby informing treatment intensity and scope.

In conclusion, this study developed and validated a multidimensional spatiotemporal heterogeneity model derived from pretreatment ^18^F-FDG PET-CT that achieved excellent predictive performance for unresectable LA-NSCLC treatment response across all cohorts, outperforming single-scale prediction models. Furthermore, the model exhibited strong clinical generalizability, maintaining robust predictive accuracy across different pathologic subtypes and treatment modalities, while also showing significant prognostic stratification power for PFS and OS. This model provides a noninvasive, precise biomarker for unresectable LA-NSCLC management, offering a theoretical foundation for personalized clinical decision making.

## Data Availability

A data sharing statement provided by the authors is available with this article at DOI https://doi.org/10.1200/PO-26-00609. The data that support the findings of this study are available from the corresponding author upon reasonable request, subject to institutional and ethical restrictions. The analysis code can be made available from the corresponding author upon reasonable request.
