# Supplementary material for: Decoding Treatment Response and Prognosis in Unresectable Locally Advanced Non–Small Cell Lung Cancer by Integrating Spatiotemporal Heterogeneity From 18F-FDG Positron Emission Tomography-Computed Tomography: A Multicenter Study
Source: JCO Precis Oncol. 2026 Oct 2;10(10):e2600609. doi: 10.1200/PO-26-00609 (PMC13657396; doi:10.1200/PO-26-00609)
Supplement: Supplementary file 1 [file po-10-e2600609-s001.pdf]

ASCO Journals Data Sharing Statement Questionnaire

**1. Manuscript Title**	Decoding Treatment Response and Prognosis in Unresectable LA-NSCLC by Integrating Spatiotemporal Heter
**2. First Author Last Name**	Ji
**3. Does your manuscript use ONLY data from a publicly available database (eg, SEER, Medicare)?**<br>If "Yes," please specify the database; no additional information is needed.<br>If "No," please complete questions 4-17.	No. The primary dataset consists of institutional patient data collected at two medical centers; public TCGA transcriptomic data were additionally used for exploratory gene analysis.
**4. Will the data collected for your study be made available to others?**<br>(If "No," enter context for your decision)	Yes. De-identified individual participant data will be made available upon reasonable request, subject to institutional and ethical restrictions.
**5. List which data are available**	De-identified individual participant data underlying the results reported in this article, including baseline clinical d
**6. Additional information about the data**<br>(Enter "None," if applicable)	Raw patient imaging data cannot be publicly shared because of patient privacy and institutional policies. The TCGA transcriptomic data used for exploratory analysis are publicly available at http://cancergenome.nih.gov/.
**7. List how or where the data can be obtained**<br>(e.g., e-mail address, URL, or other repository)	By e-mail request to the corresponding author: 17660086108@163.com (Shandong Cancer Hospital and Institute, Shandong First Medical University and Shandong Academy of Medical Sciences, Jinan, Shandong, China).
**8. List beginning and end dates that data will be available**<br>(e.g., MM/DD/YYYY to MM/DD/YYYY)	Beginning on the date of article publication, with no end date (indefinitely).
**9. List any supporting documents**<br>(Enter "None," if applicable)	Study protocol and statistical analysis code (R scripts).
**10. Enter additional information about supporting documents**<br>(Enter "None," if applicable)	None.

**11. How or where can supporting documents be obtained?** (e.g., e-mail address, URL, or other repository)	By e-mail request to the corresponding author: 17660086108@163.com.
**12. List beginning and end dates that supporting documents will be available** (e.g., MM/DD/YYYY to MM/DD/YYYY)	Beginning on the date of article publication, with no end date (indefinitely).
**13. Indicate to whom data will be available** (Enter "U/K" for unknown; "N/A" for not applicable)	Qualified researchers who submit a methodologically sound proposal for academic research purposes.
**14. Indicate for what type of analysis or purpose** (Enter "U/K" for unknown)	For any academic research purpose consistent with the approved proposal, such as external validation, meta-analysis, or secondary analyses.
**15. Indicate by what mechanism** (Enter "U/K" for unknown)	Proposals will be reviewed and approved by the corresponding author and the institutional ethics committee; data will be shared after a data use agreement is signed.
**16. Enter any other restrictions** (Enter "None," if applicable)	Only de-identified data will be shared; data sharing is subject to approval by the institutional ethics committee and compliance with patient privacy regulations.
**17. Enter any additional information** (Enter "None," if applicable)	This study was approved by the Ethics Committee of The Second Qilu Hospital of Shandong University (Approval No. KYLL2024487); the requirement for informed consent was waived due to the retrospective nature of the study.

(Optional) If you have included or would like to include a Data Sharing Statement in addition to the above, please provide on the following page.

Data Sharing Statement

The data that support the findings of this study are available from the corresponding author (17660086108@163.com) upon reasonable request, subject to institutional and ethical restrictions. Raw imaging data cannot be publicly shared because of patient privacy and institutional policies. The TCGA transcriptomic data used for exploratory gene analysis are publicly available at http://cancergenome.nih.gov/.
