# Supplementary material for: Decoding Treatment Response and Prognosis in Unresectable Locally Advanced Non–Small Cell Lung Cancer by Integrating Spatiotemporal Heterogeneity From 18F-FDG Positron Emission Tomography-Computed Tomography: A Multicenter Study
Source: JCO Precis Oncol. 2026 Oct 2;10(10):e2600609. doi: 10.1200/PO-26-00609 (PMC13657396; doi:10.1200/PO-26-00609)
Supplement: Supplementary file 2 [file po-10-e2600609-s002.pdf]

Supplementary Information

Decoding Treatment Response and Prognosis in Unresectable LA-NSCLC by Integrating Spatiotemporal Heterogeneity from ^{18}F-FDG PET/CT: A Multi-Center Study

Author List

Yu Ji, MD, PhD[1], Jiaqi Wang, BS[2], Meixia Zhou, MD[3], Yaru Wang, MD[1], Zhengjun Dai, MS[4], Jingsong Zheng, MD[5*]

Author Institutions

1. Department of Radiology, The Second Qilu Hospital of Shandong University, Jinan, Shandong, China.
2. Department of Cancer Medical Center, People's Hospital of Chengwu County, Heze, Shandong, China.
3. Department of Radiology, Shandong University, Jinan, Shandong, China.
4. Institute of Research and Clinical Innovations, Neusoft Medical Systems Co. Ltd, Beijing, China
5. Center for Nuclear Medicine and Molecular Imaging, Shandong Cancer Hospital and Institute, Shandong First Medical University and Shandong Academy of Medical Sciences, Jinan, 250117, China.

Corresponding author

Jingsong Zheng

ORCID: 0000-0002-8510-0275

Center for Nuclear Medicine and Molecular Imaging, Shandong Cancer Hospital and Institute, Shandong First Medical University and Shandong Academy of Medical Sciences, Jinan, Shandong, China.

E-mail: 17660086108@163.com

Content:

Supplementary materials

Supplementary Figures 1-10

Supplementary Tables 1-6

Treatment modalities and patient stratification

Among the 434 enrolled patients with locally advanced NSCLC (LA-NSCLC), 234 (54.0%) received definitive chemoradiotherapy as curative-intent treatment, comprising concurrent or sequential platinum-based chemotherapy combined with thoracic radiotherapy. The remaining 200 (46.0%) patients did not receive definitive chemoradiotherapy and instead received heterogeneous non-surgical regimens. Specifically, 39 (9.0%) received systemic chemotherapy alone, 81 (18.7%) received immunotherapy-based regimens (PD-1/PD-L1 inhibitors, as monotherapy or in combination with chemotherapy), 57 (13.1%) received targeted therapy (EGFR-TKI, ALK inhibitors, or other targeted agents for patients with actionable driver mutations), and 23 (5.3%) received individualized systemic regimens or clinical trial protocols due to severe comorbidities or medical frailty precluding standard treatment. No patient underwent surgical resection as the primary therapy. All patients in both groups completed at least two cycles of systemic therapy to the first radiological response assessment at 6–10 weeks.

Treatment response assessment

For patients in the definitive chemoradiotherapy group, tumor response was evaluated according to the internationally recognized Response Evaluation Criteria in Solid Tumors (RECIST) version 1.1.The specific criteria were as follows: (1) complete response (CR): disappearance of all target lesions (primary lesion and regional lymph nodes) with no new lesions; (2) partial response (PR): a sum of the diameters of the target lesions decreased by ⩾30% (using the baseline CT diameters as reference); (3) progressive disease (PD): a sum of the diameters of the target lesions increased by ⩾20%, or an absolute increase of ⩾5 mm, or the appearance of new lesions; (4) stable disease (SD): changes in the target lesions that did not meet the criteria for PR or PD. For patients in the non-chemoradiotherapy group who received combined immunotherapy, the iRECIST criteria were applied, while the RECIST 1.1 criteria were retained for those not receiving immunotherapy. The iRECIST criteria were defined as: (1) iCR/iPR/iSD definitions were consistent with RECIST 1.1, and the response had to be maintained for ⩾4 weeks; (2) Initial assessment of PD was recorded as unconfirmed progression (iUPD), which required confirmation by a follow-up scan within 8 weeks: persistent progression was defined as confirmed progression (iCPD), otherwise, the response was reclassified based on the current best response. All response evaluations were independently and blindly assessed by two senior radiologists. In cases of disagreement, a third senior radiologist was consulted to reach a final consensus on the tumor's treatment response. Patient follow-up adhered to the standard oncology surveillance protocol, typically conducted every 3 to 6 months for the first 3 years, every 6 months for years 4 to 5, and annually thereafter. Progression-free survival (PFS) and overall survival (OS) were designated as the secondary and tertiary endpoints of the study, respectively. PFS was defined as the time from diagnosis to disease progression of any cause or death. OS was defined as the time from diagnosis to death from any cause or to the last follow-up.

^{18}F-FDG PET/CT image acquisition and preprocessing

All ^{18}F-FDG PET/CT image acquisitions using Philips Ingenuity TF (Netherlands), Philips Big

Bore TF (Netherlands), and Siemens Biograph Horizon (Germany) scanner systems. All multicenter ^{18}F-FDG PET/CT images included in this study were acquired in compliance with the standards of the European Association of Nuclear Medicine (EANM) Research Ltd Fluorine-18 accreditation specifications (EARL1 or EARL2) to ensure quantitative consistency. The radioactive tracer ^{18}F-FDG was supplied by Nanjing Anke Co., Ltd. and Beijing Atom High Tech Co., Ltd., or produced by a GE MINItrace cyclotron. The radiochemical purity of all administered ^{18}F-FDG exceeded 95%, with a pH range of 5.0–7.0, complying with the quality control standards for radioactive pharmaceuticals set by the International Atomic Energy Agency (IAEA). All scans were performed within one week prior to the initiation of antitumor therapy. Building upon this foundation, the present study implemented a four-step standardized preprocessing pipeline to mitigate scanner- and protocol-related biases arising from multicenter PET/CT imaging data. Detailed scanning procedures, image preprocessing, and tumor segmentation methods are provided in the Supplementary Material.

The standardized PET/CT scanning protocol was strictly followed for all enrolled patients. Patients were required to fast for at least 6 hours and have their pre-scan blood glucose levels controlled below 11.1 mmol/L (200 mg/dL). After intravenous injection of ^{18}F-FDG at a dose of 4.4–5.5 MBq/kg, patients rested in a quiet, dimly lit room for 50–60 minutes to allow for adequate biodistribution of the radiopharmaceutical. During this uptake period, patients were instructed to avoid speaking, chewing, or any physical activity to minimize physiological muscular uptake of ^{18}F-FDG. For the imaging procedure, patients were positioned supine on the scanner table with their arms raised above their head. The scanning range covered from the skull vertex to the upper-mid femur. Imaging commenced with a low-dose spiral CT (LDCT) scan acquired for attenuation correction and anatomical localization, using parameters typically set at 120 kVp and 300 mA·s tube current. Immediately following the CT scan, a multi-station PET emission scan was performed in the same position, typically involving 7–9 bed positions with an acquisition time of approximately 1–2 minutes per bed position. The PET images were reconstructed using the CT data for attenuation correction, and the corrected PET images were then fused with the corresponding CT images to generate coronal and sagittal fusion images for diagnostic interpretation.

^{18}F-FDG PET/CT image preprocessing

A four-step standardized preprocessing pipeline was implemented to mitigate scanner- and protocol-related biases arising from multicenter PET/CT imaging data. Firstly, the attenuation-corrected PET raw data were converted into standardized uptake value (SUV) maps using the formula: SUV = voxel activity (kBq/mL) / [injected dose (MBq) × body weight (kg)], in accordance with EANM guidelines for quantitative oncologic imaging. Concurrently, CT images were uniformly adjusted to lung window settings to standardize soft-tissue and pulmonary parenchyma contrast. Subsequently, under the constraint of alignment at the tumor's largest axial cross-section, multimodal PET/CT registration was performed using Elastix (v5.0.1) software based on rigid transformation. The spatial alignment was optimized by maximizing the Dice similarity coefficient, and all registration results were quality-controlled by an experienced radiologist; suboptimal alignments were refined using 3D Slicer (v4.11) to ensure accuracy for subsequent habitat analysis. Thereafter, the registered PET and CT images underwent voxel intensity normalization and were resampled to an isotropic resolution of 1 × 1 × 1 mm^3 using bilinear interpolation, thereby eliminating intersite variability in voxel dimensions. Finally, to reduce radiomic feature sensitivity to noise, both CT and PET images underwent intensity discretization with bin widths of 25

Hounsfield units and 0.1 SUV, respectively, generating discrete grayscale histograms for robust feature extraction.

Tumor segmentation

The specific tumor segmentation workflow was as follows: initially, the anatomical boundaries of the tumor were defined on CT images using lung window settings (window width: 1500 HU; window level: -600 HU). In cases where CT alone was insufficient to accurately delineate tumor margins—such as when adjacent pulmonary atelectasis was present or when the tumor abutted the mediastinum or chest wall—PET images were integrated to assist in boundary determination. The segmentation workflow in this study was designed to ensure high consistency and accuracy through a multi-stage process. First, one radiologist (with 5 years of clinical experience) completed the initial tumor segmentation using 3D Slicer software. Then, two senior radiologists (each with more than 10 years of clinical experience) independently reviewed the initial segmentation results. For any discrepancies, the three radiologists conducted a joint discussion to reach a consensus and made necessary adjustments to the initial segmentation mask. The reproducibility of segmentation and derived features was evaluated in 30 randomly selected cases by assessing both intra-observer (4-week interval) and inter-observer (independent radiologist) agreement using intraclass correlation coefficients (ICCs). Only features demonstrating good consistency (ICC ≥ 0.75) in both assessments were retained for subsequent analysis. Following segmentation, four key metabolic parameters—SUVmax, SUVmean, MTV, and TLG—were quantified within the entire tumor and each distinct habitat using the PET IndiC plugin in 3D Slicer.

Radiomics feature extraction

The extracted features comprised four categories: (1) Shape features (n=14). (2) First-order statistical features (n=18). (3) Texture features: Gray-Level Co-occurrence Matrix (GLCM, n=22), Gray-Level Run-Length Matrix (GLRLM, n=16), Gray-Level Size Zone Matrix (GLSZM, n=16), Gray-Level Dependence Matrix (GLDM, n=14), and Neighboring Gray-Tone Difference Matrix (NGTDM, n=5). (4) Filtered features (n=905): Generated by applying nine distinct filters—Laplacian of Gaussian (LoG), Wavelet, Square, Square Root, Logarithm, Exponential, Gradient, Local Binary Pattern (3D), and Local Binary Pattern (2D)—to enhance specific image patterns. First-order and texture features were then extracted from these filtered VOIs. Collectively, this integrated approach leveraging dual-modality PET/CT metabolic-anatomical information established a high-dimensional quantification system comprising 10,100 radiomic features: 2,020 traditional whole-tumor radiomics features (1,010 features × 2 modalities) and 8,080 habitat features (1,010 features × 2 modalities × 4 habitat subregions).

This study encountered a class imbalance in the enrolled LA-NSCLC cohort, with the treatment responsive group constituting 63.8% of patients and the non-responsive group 36.2%. Such an imbalance could predispose the model to bias towards the majority class, potentially reducing its sensitivity in predicting treatment failure. To address this, the Synthetic Minority Over-sampling Technique (SMOTE) was applied during the training phase to achieve a 1:1 class balance. It is crucial to note that all synthetically generated samples were used exclusively for model training, while the internal and external validation sets were strictly maintained in their original, unaltered state. This approach ensures that the evaluation of model performance accurately reflects its generalizability in a real-world clinical scenario. To manage the high dimensionality of multi-modal radiomics features and identify the most discriminative habitat features, a four-stage feature selection pipeline was implemented. Initially, the Mann-Whitney U test (two-tailed, α=0.05) was

employed for preliminary screening, retaining features with a significance level of $P < 0.05$. Subsequently, features exhibiting high redundancy were eliminated based on Spearman's rank correlation coefficient; if the absolute correlation coefficient between any two features exceeded 0.9, only the feature with the stronger association to the outcome variable was retained. The third step utilized the Maximum Relevance and Minimum Redundancy (mRMR) algorithm to select a subset of the top 100 features that were highly relevant to treatment response while maintaining low inter-feature correlation. Finally, the Least Absolute Shrinkage and Selection Operator (LASSO) regression algorithm, coupled with 10-fold cross-validation, was applied. By employing L1 regularization, LASSO shrunk the coefficients of less informative features to zero, ultimately selecting a parsimonious set of non-zero coefficient features for model construction. This comprehensive methodology effectively mitigates the risk of overfitting and facilitates the development of a robust and interpretable predictive model by capturing the most critical habitat radiomics features associated with treatment efficacy.

Calculation of time-heterogeneous parameters NHOC and NHOP

The standardized hotspot-to-centroid distance (NHOC) was defined as the three-dimensional Euclidean distance from the point of maximum metabolic activity (i.e., the FDG uptake hotspot) within the tumor to its geometric centroid, normalized by the radius of an equivalent sphere of identical volume. Similarly, the standardized hotspot-to-periphery distance (NHOP) was defined as the shortest distance from the metabolic hotspot to the nearest tumor boundary, also normalized by the equivalent sphere radius. Based on the method used to characterize metabolic activity, each parameter was further subdivided into two variants: NHOCmax/NHOPmax, calculated using the single voxel with the highest metabolic value (SUVmax); and NHOCpeak/NHOPpeak, calculated based on the centroid of a local high-metabolism region, defined as the center of a 1 cm^3 spherical volume yielding the maximum average SUV (SUVpeak). For tumors with a diameter less than 12 mm (deemed too small to reliably define a spherical volume of interest), SUVmax was automatically used instead of SUVpeak. The specific calculation formulas were as follows:

1. Equivalent sphere radius R (to eliminate the influence of tumor volume):

$$\boldsymbol{R = \sqrt[3]{\frac{3 \times MTV}{4\pi}}}$$

2. 3D Euclidean Distance (Foundation for Spatial Position Calculation):

$$\boldsymbol{d(A,B) = \sqrt{(xA - xB)^2 + (yA - yB)^2 + (zA - zB)^2}}$$

3. Normalized Time Heterogeneity Parameters:

$$\boldsymbol{NHOC_{max} = \frac{d(SUV_{max}, Centroid)}{R}}$$

$$\boldsymbol{NHOC_{peak} = \frac{d(SUV_{peak}, Centroid)}{R}}$$

$$\boldsymbol{NHOP_{max} = \frac{min(d(SUV_{max}, Perimeter)}{R}}$$

$$\boldsymbol{NHOP_{peak} = \frac{min(d(SUV_{peak}, Perimeter)}{R}}$$

To exclude potential confounding by tumor volume, we performed a collinearity assessment. The Pearson correlation coefficient between NHOP-SUVmax and MTV was negligible ($r = 0.089$,

P = 0.192). Furthermore, the variance inflation factor (VIF) for NHOP-SUVmax in the multivariate logistic regression model was 1.008, essentially equal to the ideal value of 1.0 and far below the conventional collinearity threshold of 5, confirming the absence of collinearity and the size-independence of this parameter.

Treatment response related gene analysis

To mitigate batch effects between the LUAD and LUSC transcriptomic datasets, we applied the ComBat function from the sva package to harmonize the data. Subsequently, differential expression analysis was performed using the limma package to identify genes that were significantly differentially expressed between the responsive and non-responsive groups, with a threshold of $|\log_2(\text{fold change})| \geq 1.5$ and an adjusted P-value < 0.01. A glycolytic gene set was retrieved from the Molecular Signatures Database (MSigDB), and intersected with the therapy-response-related differentially expressed genes to identify glucose metabolism-related genes associated with treatment outcome. In addition, gene set enrichment analysis (GSEA) was employed to systematically interpret the biological functions and pathway regulation mechanisms of the differentially expressed genes. Specifically, GSEA was conducted based on the KEGG (Kyoto Encyclopedia of Genes and Genomes) functional database to evaluate enrichment trends of the differentially expressed genes across various functional pathways. The "c2.cp.kegg.v7.5.1.symbols.gmt" gene set from MSigDB was used as the background annotation file. The expression matrix was rank-ordered and analyzed using GSEA software with 1,000 phenotype permutations. Pathways with a normalized enrichment score, nominal P-value < 0.05, and false discovery rate < 0.25 were considered significantly enriched.

Statistical analysis

Continuous variables were classified as normally or non-normally distributed based on the Kolmogorov-Smirnov test. Normally distributed variables were expressed as mean ± standard deviation (Mean ± SD), while non-normally distributed variables were summarized as median and interquartile range (Median [IQR]). Categorical variables were presented as frequencies and percentages (n (%)). Group comparisons were conducted using a stratified testing strategy: continuous variables were analyzed with independent t-tests (upon confirmation of homogeneity of variances by Levene's test) or the Mann–Whitney U test for non-parametric data; categorical variables were assessed using Pearson's chi-square test when frequencies were ⩾5, and Fisher's exact test when frequencies were <5. All hypothesis tests were two-sided, with a significance threshold set at P < 0.05, and key results were reported with point estimates and 95% confidence intervals.

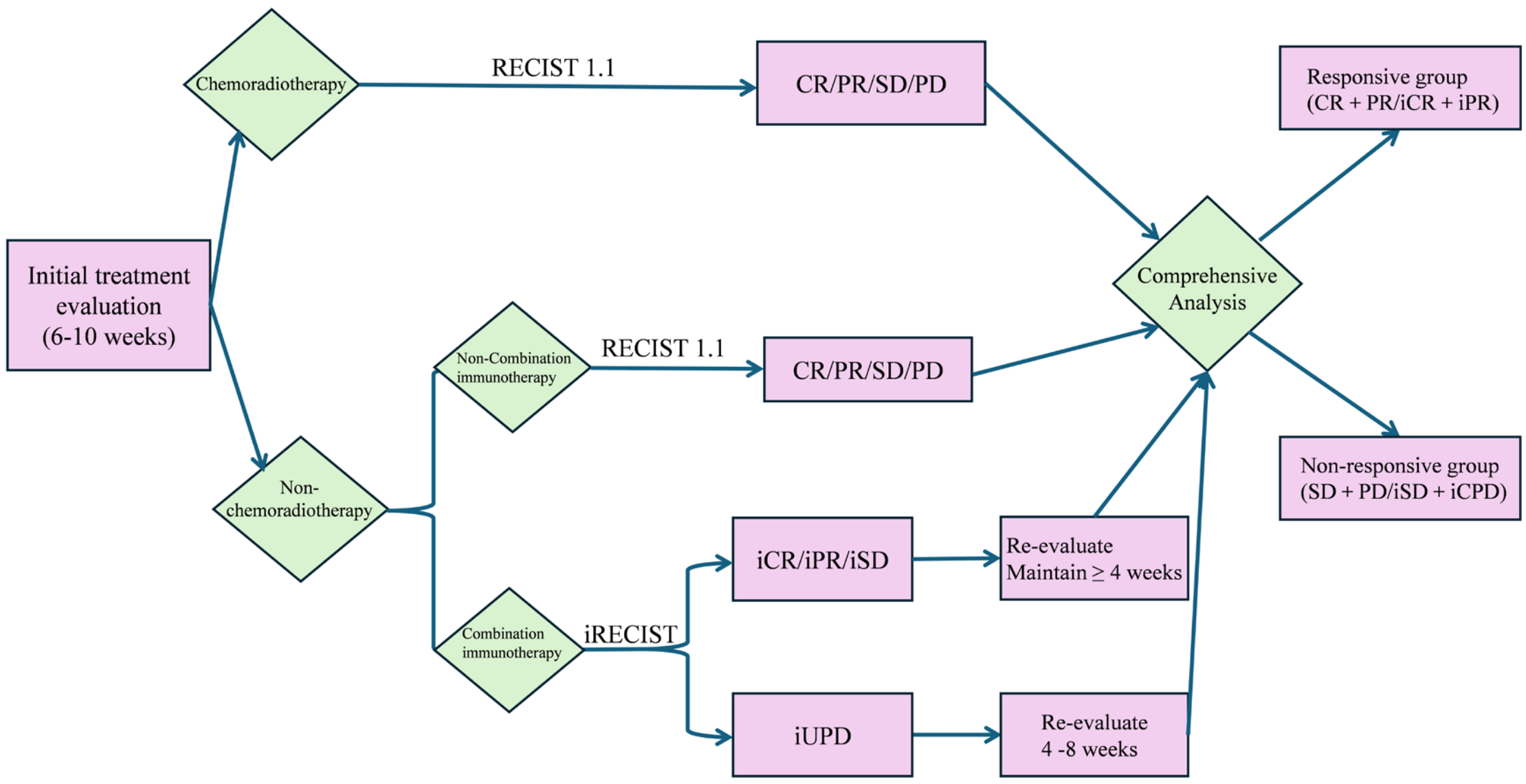

Supplementary Fig 1. Schematic diagram of treatment response evaluation.

CR = complete response, PR = partial response, SD = stable disease, PD = progressive disease, iCR = immunotherapy complete response, iPR = immunotherapy partial response, iSD = immunotherapy stable disease, iUPD = immunotherapy unconfirmed progression, iCPD = immunotherapy confirmed progression.

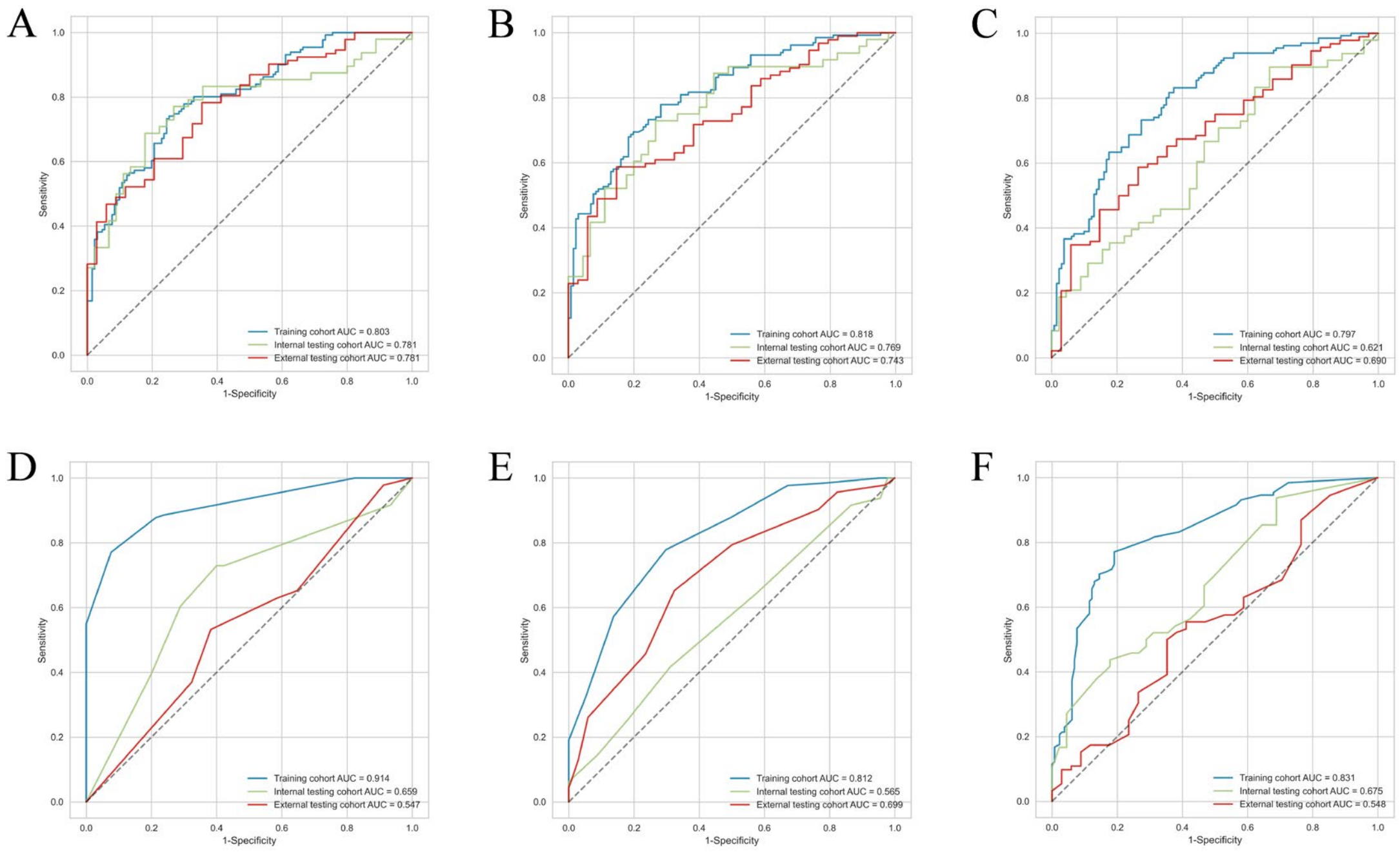

Supplementary Fig 2. Comparison of receiver operating characteristic curves among different machine learning models.

The ROC curve shows the predictive performance of LR (A), SVM (B), NB (C), DT (D), KNN (E), AdaBoost (F) models on treatment response in LA-NSCLC. Source data are provided in Supplementary Table 2.

LR = logistic regression, SVM = support vector machine, NB = naive bayes, KNN = k-nearest neighbors, DT = decision tree, AdaBoost = adaptive boosting.

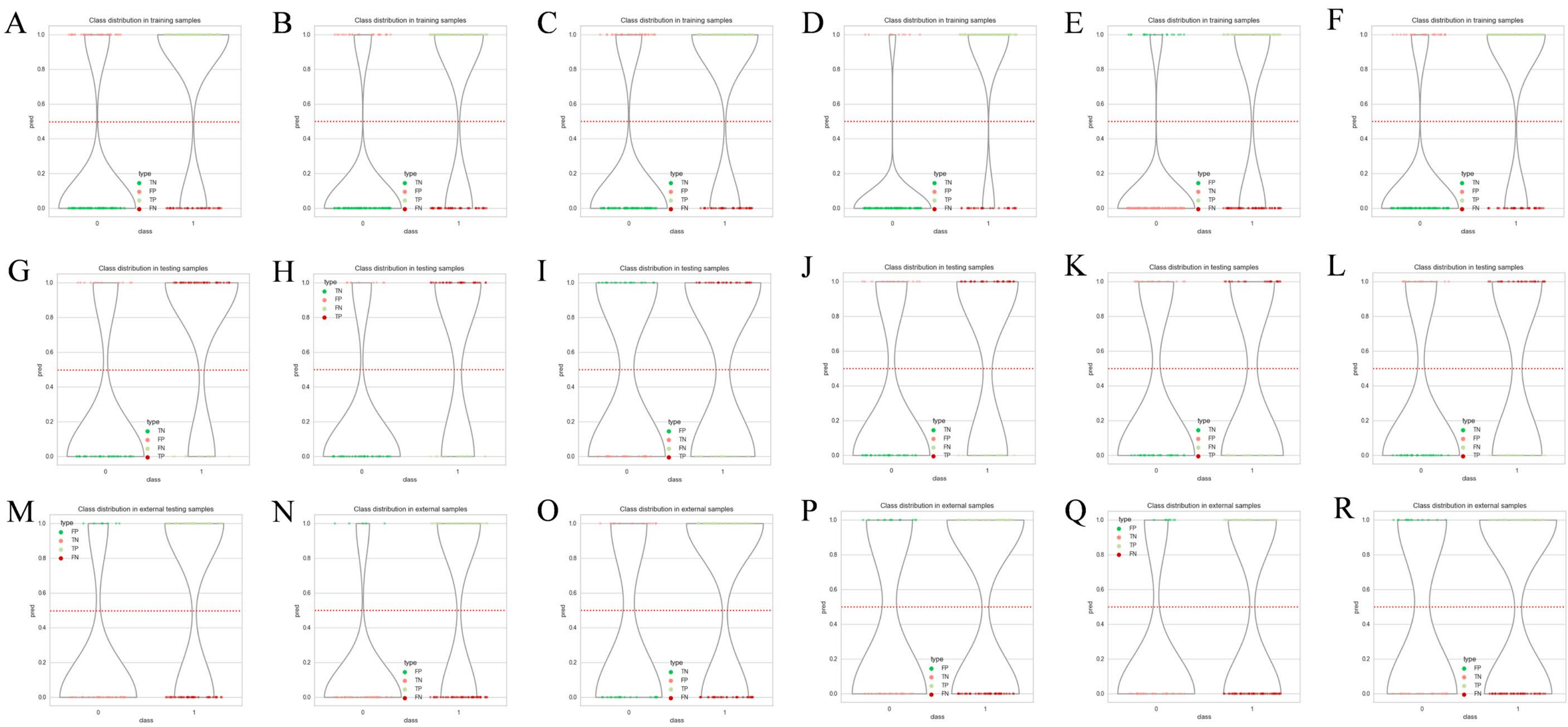

Supplementary Fig 3. Visualize the distribution of multiple machine learning models' prediction results.

Performance visualization of six machine learning models for treatment response prediction: LR (A, G, M), SVM (B, H, N), NB (C, I, O), DT (D, J, P), KNN (E, K, Q), and AdaBoost (F, L, R). Results are shown for the training (A-F), internal validation (G-L), and external validation sets (M-R).

LR = logistic regression, SVM = support vector machine, NB = naive bayes, KNN = k-nearest neighbors, DT = decision tree, AdaBoost = adaptive boosting, TN = True Negative, FP = False Positive, FN = False Negative, TP = True Positive.

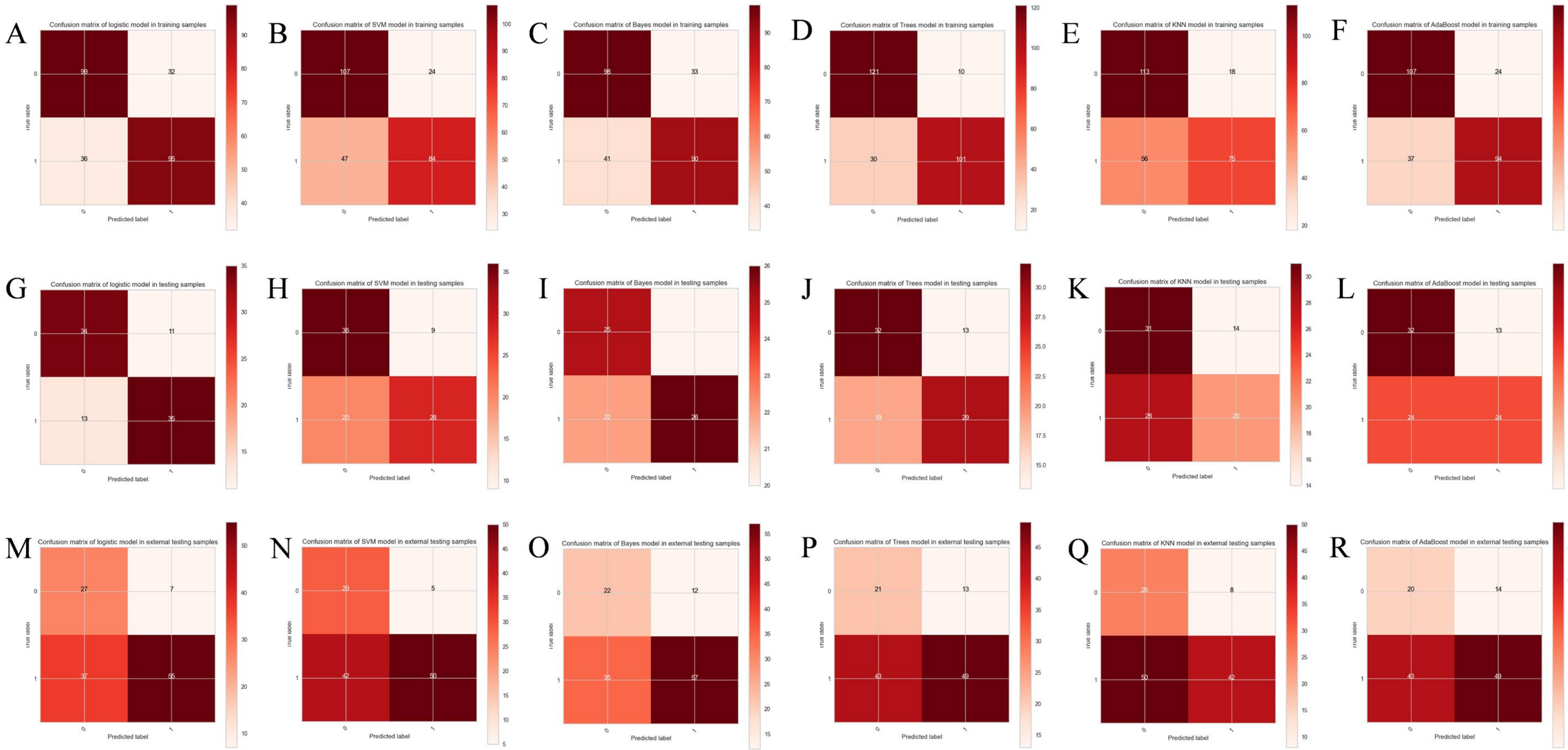

Supplementary Fig 4. Confusion matrix of multiple machine learning prediction models.

Confusion matrices showing the performance of six machine learning models in predicting treatment response across different datasets: LR (A, G, M); SVM (B, H, N); NB (C, I, O); DT (D, J, P); KNN (E, K, Q); and AdaBoost (F, L, R). Results are shown for the training (A-F), internal validation (G-L), and external validation sets (M-R).

LR = logistic regression, SVM = support vector machine, NB = naive bayes, KNN = k-nearest neighbors, DT = decision tree, AdaBoost = adaptive boosting.

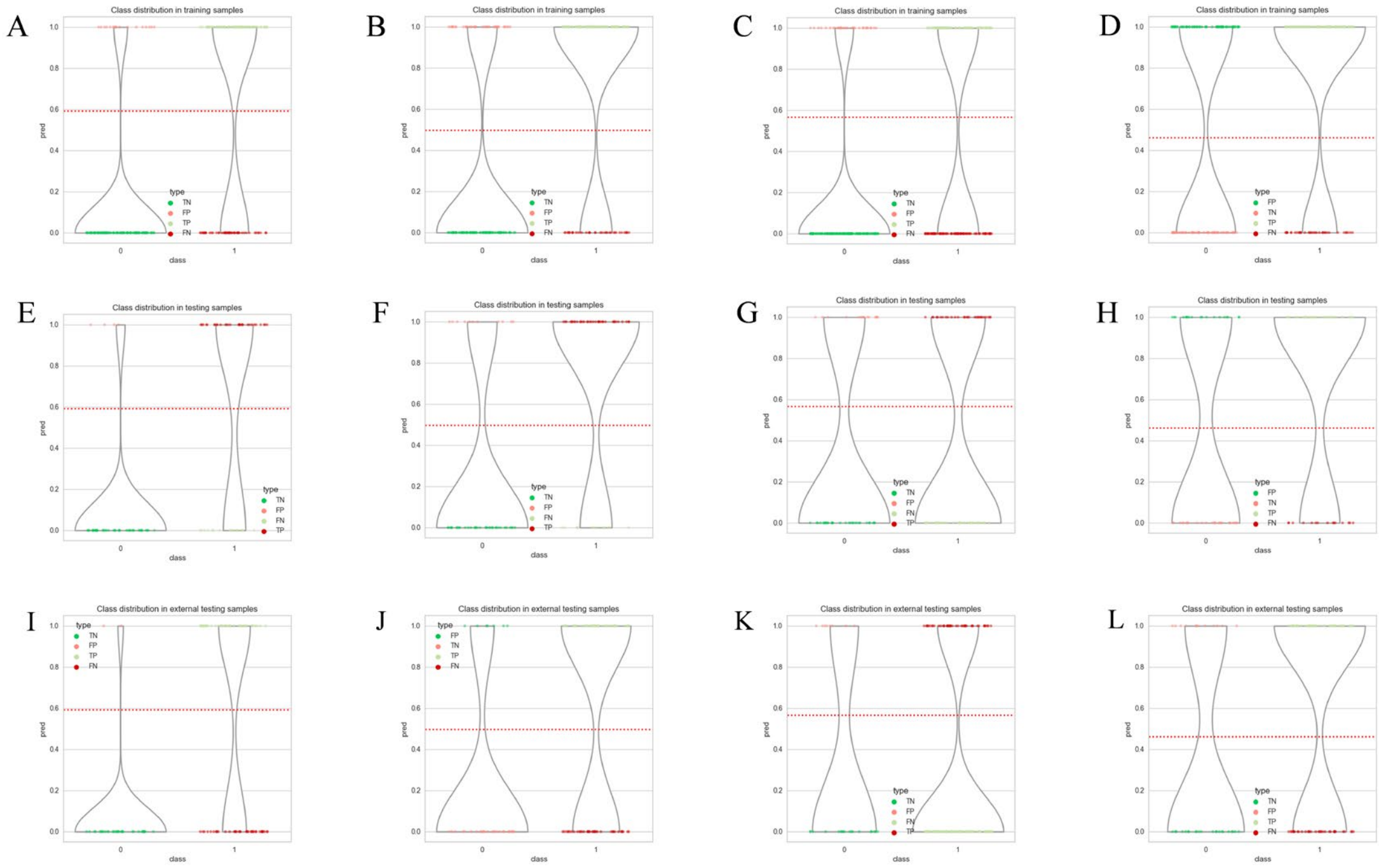

Supplementary Fig 5. Visualize the distribution of multiple models' prediction results.

Performance visualization of four models for treatment response prediction: spatiotemporal heterogeneity model (A, E, I), habitat model (B, F, J), whole-tumor radiomics model (C, G, K), and clinical/metabolic models (D, H, L). Results are shown for the training (A-D), internal validation (E-H), and external validation sets (I-L).

TN = True Negative, FP = False Positive, FN = False Negative, TP = True Positive.

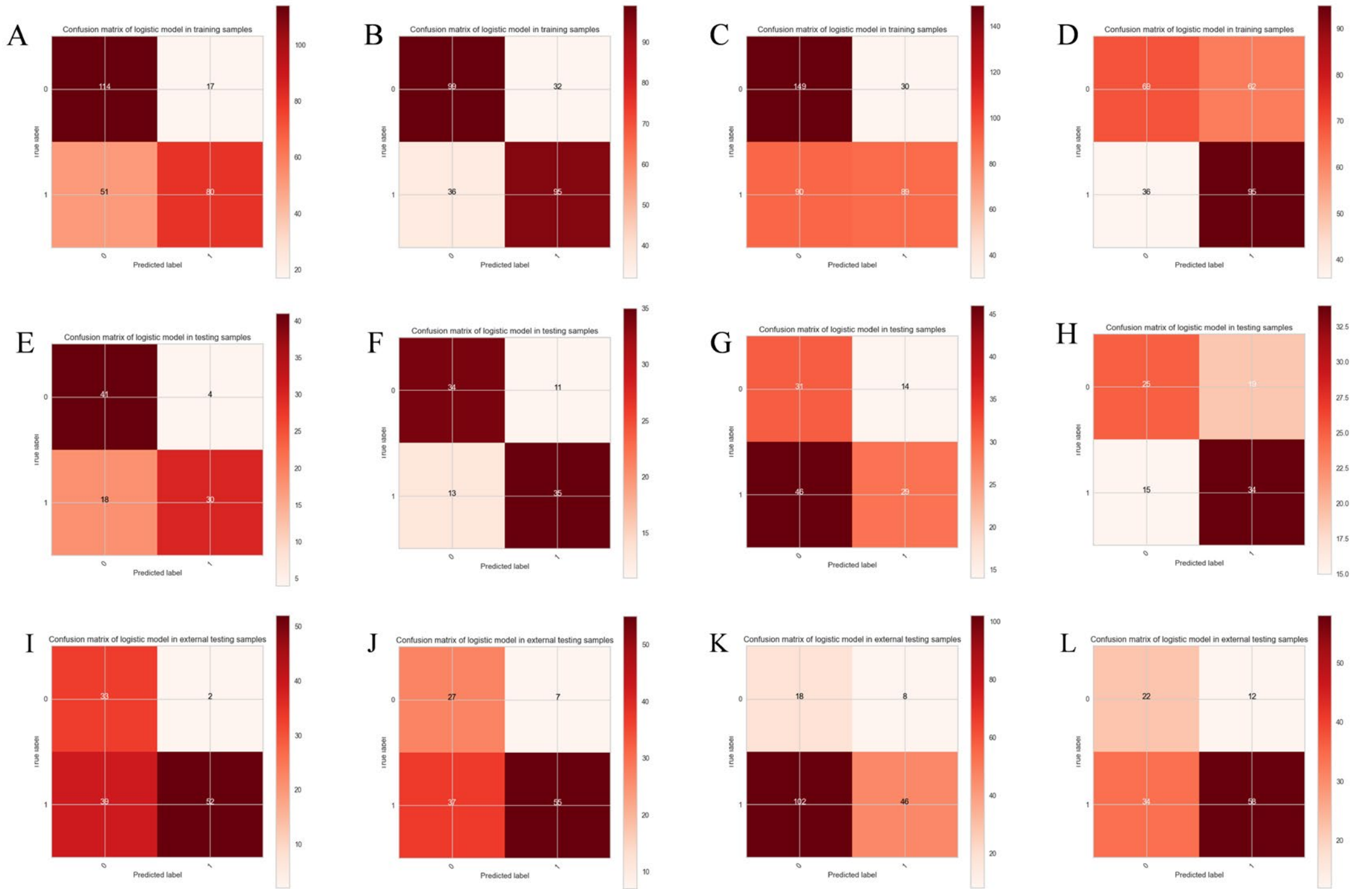

Supplementary Fig 6. Confusion matrix of multiple prediction models.

Confusion matrices showing the performance of four machine learning models in predicting treatment response across different datasets: spatiotemporal heterogeneity model (A, E, I), habitat model (B, F, J), whole-tumor radiomics model (C, G, K), and clinical/metabolic models (D, H, L). Results are shown for the training (A-D), internal validation (E-H), and external validation sets (I-L).

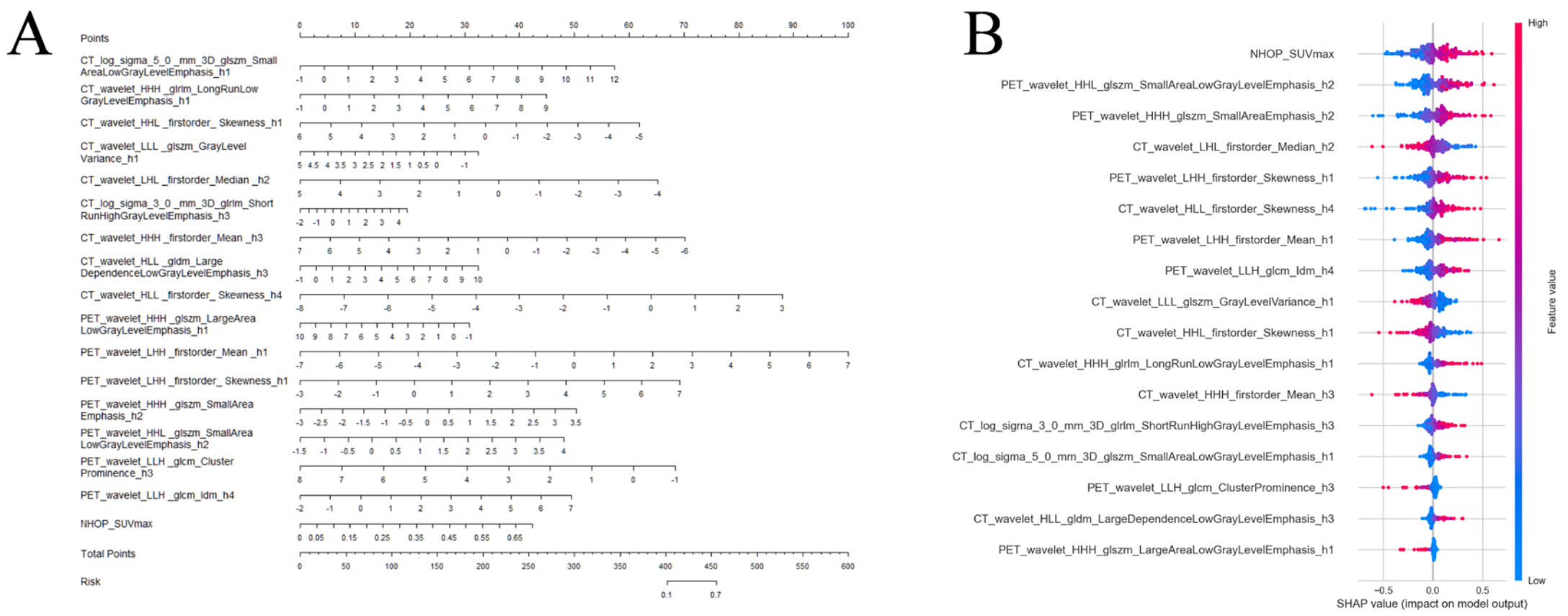

Supplementary Fig 7. Model interpretation.

Nomogram (A) for visualizing prediction rules. SHAP summary plot (B) showing global feature importance.

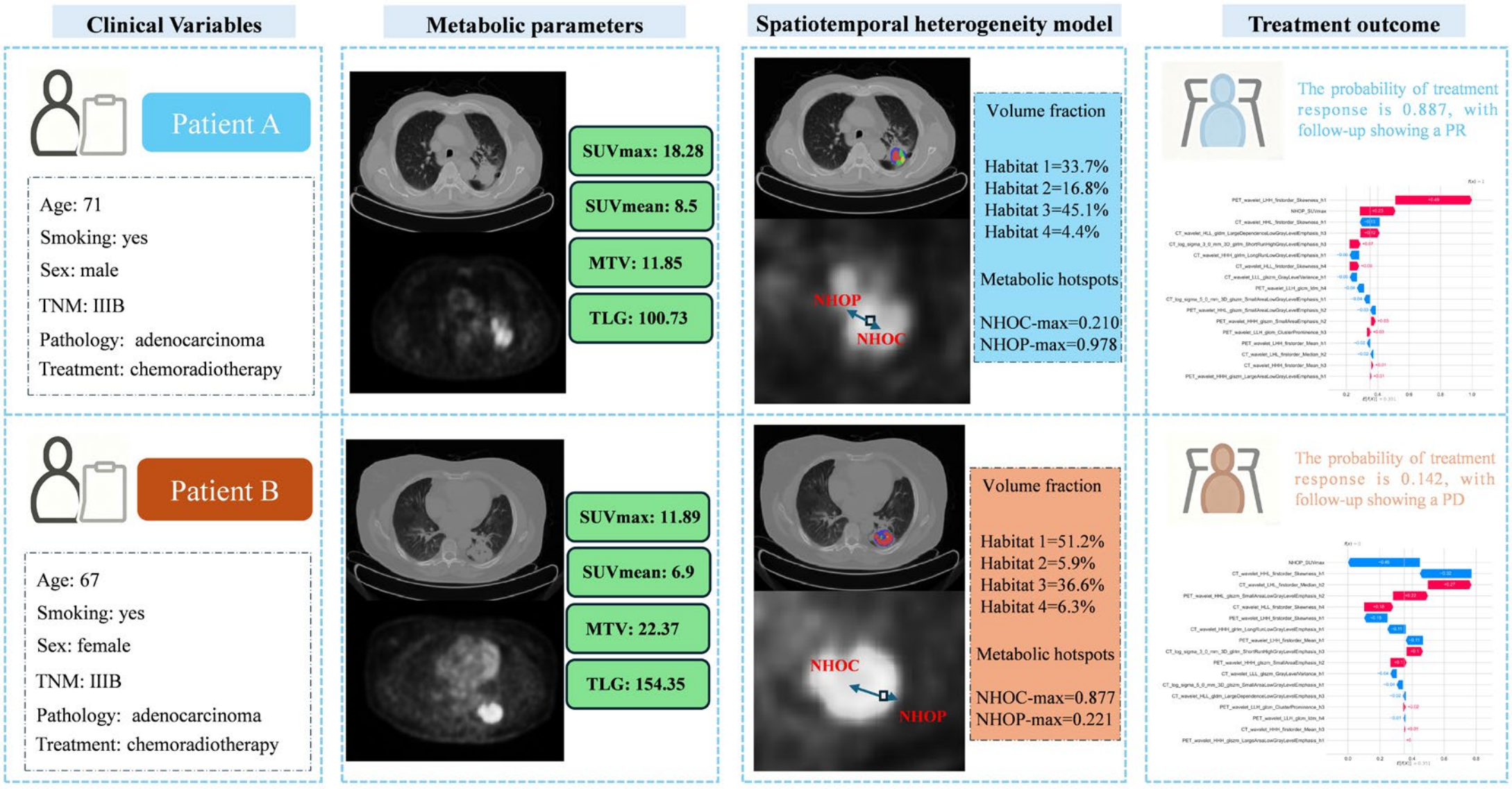

Supplementary Fig 8. Spatiotemporal Heterogeneity Model for Clinical Application in Representative Patients.

Spatiotemporal heterogeneity model for treatment response prediction in two representative patients with unresectable LA-NSCLC. Although Patients A and B presented with similar baseline clinical characteristics, conventional metabolic evaluation indicated that Patient A had greater metabolic activity and tumor burden than Patient B; nevertheless, the model assigned them to opposing risk categories. For each patient, spatial habitat maps were generated from PET and CT images using the Otsu algorithm, followed by habitat feature extraction and volume fraction calculation; temporal heterogeneity parameters (NHOC and NHOP) were derived from metabolic hotspot displacement. Patient A was stratified as low-risk, with a predominant PETlow-CTlow habitat (Habitat 2, volume fraction 16.8%) and a centrally located hotspot (NHOC-max 0.210, NHOP-max 0.978), yielding an 88.7% predicted probability of response, consistent with the subsequent partial response. In contrast, Patient B was stratified as high-risk, with a predominant PEThigh-CThigh habitat (Habitat 1, volume fraction 51.2%) and a peripherally displaced hotspot (NHOC-max 0.877, NHOP-max 0.221), yielding a 14.2% predicted probability of response, consistent with the subsequent progressive disease. Individual SHAP force plots for each patient illustrate the directional contribution of key features to the final prediction, enhancing model transparency and clinical interpretability

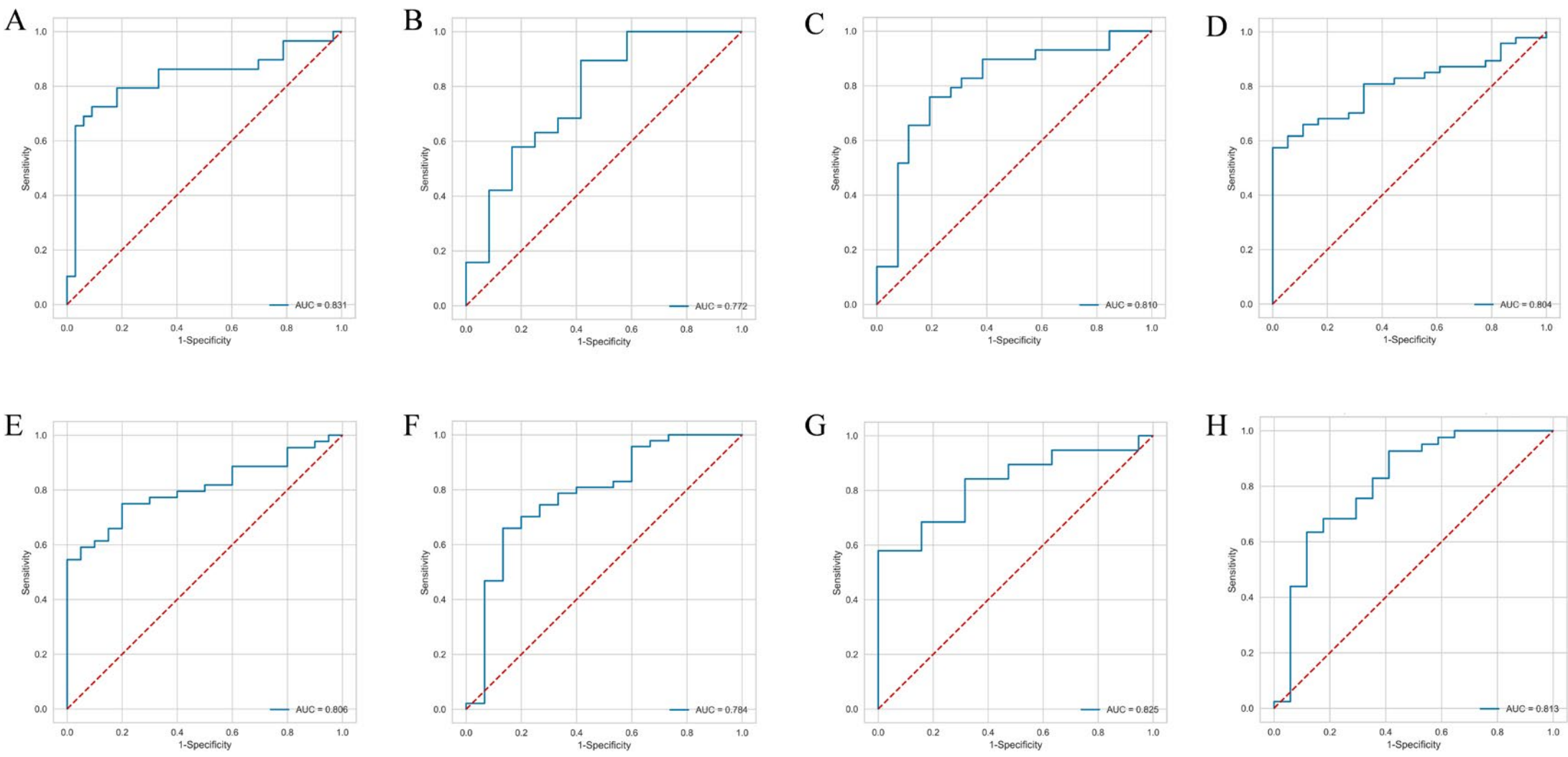

Supplementary Fig 9. Subgroup analysis of spatiotemporal heterogeneity prediction models. A and E represent the lung squamous cell carcinoma subgroup; B and F, the lung adenocarcinoma subgroup; C and G, the definitive chemoradiotherapy subgroup; and D and H, the non-chemoradiotherapy subgroup. Receiver operating characteristic (ROC) curves for the spatiotemporal heterogeneity model are shown in the internal validation set (A–D) and the external validation set (E–H).

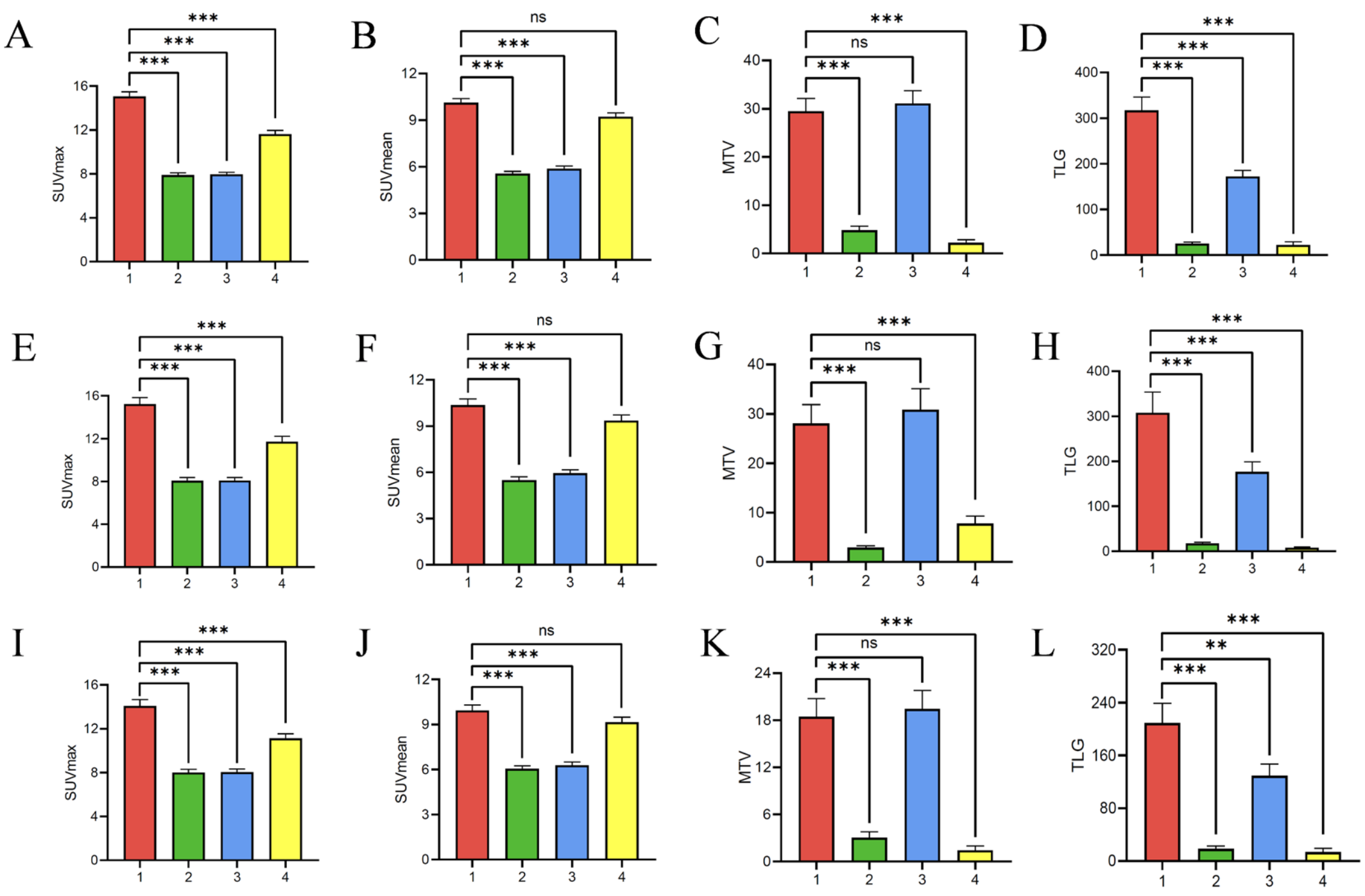

Supplementary Fig 10. Analysis of metabolic parameters across habitats.

Comparative analysis of metabolic parameters across the four habitats was conducted in the training (A-D), internal validation (E-H), and external validation (I-L) sets. Habitat 1 consistently exhibited the highest metabolic activity, demonstrating the greatest SUVmax (A, E, I) and TLG (D, H, L) values, which significantly surpassed those of all other habitats. Additionally, SUVmean was significantly elevated in Habitats 1 and 4 compared to Habitats 2 and 3 (B, F, J). In contrast, MTV values were significantly higher in Habitats 1 and 3 relatives to Habitats 2 and 4 (C, G, K). The four habitats were characterized as follows: Habitat 1 (red), PEThigh-CThigh; Habitat 2 (green), PETlow-CTlow; Habitat 3 (blue), PETlow-CThigh; and Habitat 4 (yellow), PEThi

Supplementary Table 1. Univariate and multivariate analyses related to treatment response.

Variable	Univariate analysis			Multivariate analysis		
	**OR**	**95% CI**	***P* value**	**OR**	**95% CI**	***P* value**
Age	0.995	0.974-1.017	0.660			
Sex	1.345	0.807-2.243	0.256			
T-stage	0.825	0.679-1.003	0.054			
N-stage	1.029	0.812-1.304	0.815			
Clinical-stage	0.793	0.606-1.039	0.092			
Smoking	0.905	0.589-1.390	0.648			
Tumor location	0.980	0.658-1.460	0.921			
Lung lobe involvement	1.090	0.954-1.245	0.205			
CEA	0.887	0.601-1.310	0.547			
NSE	0.916	0.616-1.363	0.666			
CYFRA21_1	0.838	0.560-1.256	0.392			
ProGRP	0.669	0.403-1.111	0.120			
SCCA	0.712	0.419-1.207	0.207			
Pathology	1.050	0.600-1.838	0.864	1.090	0.607-1.956	0.773
Treatment method	1.495	0.862-2.596	0.153	1.498	0.847-2.651	0.165
SUVmax	1.000	1.000-1.000	0.642			
SUVpeak	1.000	1.000-1.001	0.641			
SUVmean	1.000	0.999-1.001	0.644			
TLG	1.000	1.000-1.000	0.624			
MTV	0.997	0.995-1.000	0.033	0.997	0.994-1.000	0.098
NHOC-SUVmax	0.606	0.277-1.324	0.209			
NHOC-SUVpeak	0.475	0.220-1.023	0.057			
NHOP-SUVmax	14.896	4.003-55.428	＜0.001	14.058	3.611-54.733	＜0.001
NHOP-SUVpeak	4.388	1.160-16.595	0.029	1.283	0.257-6.405	0.762

CEA = carcinoembryonic antigen, NSE=neuron specific enolase, CYFRA21-1 = soluble fragment of cytokeratin 19, ProGRP = pro-gastrin-releasing peptide, SCCA = squamous cell carcinoma antigen, SUV = standardized uptake value, MTV = metabolic tumor volume, TLG = total lesion glycolysis, NHOC = normalized distance from the hotspot to the tumor centroid, NHOP = normalized distance from the hotspot to the tumor periphery.

Supplementary Table 2. The treatment response-related metabolic habitat radiomics features and interpretation.

Habitat subregion	Radiomics feature	Feature interpretation
**Habitat 1**	CT_log_sigma_5_0_mm_3D_glszm_SmallAreaLowGrayLevelEmphasis_h1	The proportion in the image of the joint distribution of smaller size zones with lower gray-level values
	CT_wavelet_HHH_glrlm_LongRunLowGrayLevelEmphasis_h1	The joint distribution of long run lengths with lower gray-level values
	CT_wavelet_HHL_firstorder_Skewness_h1	The asymmetry of the distribution of values about the Mean value
	CT_wavelet_LLL_glszm_GrayLevelVariance_h1	The variance in gray level intensities for the zones
	PET_wavelet_HHH_glszm_LargeAreaLowGrayLevelEmphasis_h1	The proportion in the image of the joint distribution of larger size zones with lower gray-level values
	PET_wavelet_LHH_firstorder_Mean_h1	The average gray level intensity within the ROI
	PET_wavelet_LHH_firstorder_Skewness_h1	The asymmetry of the distribution of values about the Mean value
**Habitat 2**	CT_wavelet_LHL_firstorder_Median_h2	The median gray level intensity within the ROI
	PET_wavelet_HHH_glszm_SmallAreaEmphasis_h2	The distribution of small size zones, with a greater value indicative of more smaller size zones and more fine textures
	PET_wavelet_HHL_glszm_SmallAreaLowGrayLevelEmphasis_h2	The proportion in the image of the joint distribution of smaller size zones with lower gray-level values
**Habitat 3**	CT_log_sigma_3_0_mm_3D_glrlm_ShortRunHighGrayLevelEmphasis_h3	The joint distribution of shorter run lengths with higher gray-level values
	CT_wavelet_HHH_firstorder_Mean_h3	The average gray level intensity within the ROI
	CT_wavelet_HLL_gldm_LargeDependenceLowGrayLevelEmphasis_h3	The joint distribution of large dependence with lower gray-level values
	PET_wavelet_LLH_glcm_ClusterProminence_h3	The skewness and asymmetry of the GLCM. A higher values implies more asymmetry about the mean while a lower value indicates a peak near the mean value and less variation about the mean
**Habitat 4**	CT_wavelet_HLL_firstorder_Skewness_h4	The asymmetry of the distribution of values about the Mean value
	PET_wavelet_LLH_glcm_Idm_h4	The local homogeneity of an image

Supplementary Table 3. The performance comparison of all models across study sets.

Set	Model	AUC (95% CI)	Accuracy	F1 score	Recall	Precision	Sensitivity	Specificity	Positive prediction	Negative prediction
Train set										
	LR	0.803 (0.758, 0.845)	0.740	0.736	0.725	0.748	0.725	0.756	0.748	0.733
	SVM	0.818 (0.776, 0.859)	0.729	0.703	0.641	0.778	0.641	0.817	0.778	0.695
	NB	0.797 (0.750, 0.841)	0.718	0.709	0.687	0.732	0.687	0.748	0.732	0.705
	DT	0.914 (0.885, 0.940)	0.847	0.835	0.771	0.910	0.771	0.924	0.910	0.801
	KNN	0.812 (0.767, 0.852)	0.718	0.670	0.573	0.806	0.573	0.863	0.806	0.669
	AdaBoost	0.831 (0.788, 0.871)	0.767	0.755	0.718	0.797	0.718	0.817	0.797	0.743
	Whole-tumor	0.673 (0.625, 0.718)	0.665	0.597	0.497	0.748	0.497	0.832	0.748	0.623
	Clinical/Metabolic	0.658 (0.600, 0.710)	0.626	0.660	0.725	0.605	0.725	0.527	0.605	0.657
	Spatiotemporal heterogeneity	0.822 (0.778, 0.862)	0.740	0.702	0.611	0.825	0.611	0.870	0.825	0.691
Internal validation set										
	LR	0.781 (0.698, 0.860)	0.742	0.745	0.729	0.761	0.729	0.756	0.761	0.723
	SVM	0.769 (0.689, 0.846)	0.688	0.659	0.583	0.757	0.583	0.800	0.757	0.643
	NB	0.621 (0.525, 0.712)	0.548	0.553	0.542	0.565	0.542	0.556	0.565	0.532
	DT	0.659 (0.566, 0.751)	0.656	0.644	0.604	0.690	0.604	0.711	0.690	0.627
	KNN	0.565 (0.467, 0.658)	0.548	0.488	0.417	0.588	0.417	0.689	0.588	0.525
	AdaBoost	0.675 (0.586, 0.765)	0.602	0.565	0.500	0.649	0.500	0.711	0.649	0.571
	Whole-tumor	0.611 (0.520, 0.696)	0.500	0.492	0.387	0.674	0.387	0.689	0.674	0.403
	Clinical/Metabolic	0.647 (0.549, 0.740)	0.634	0.667	0.694	0.642	0.694	0.568	0.642	0.625
	Spatiotemporal heterogeneity	0.821 (0.746, 0.892)	0.763	0.732	0.625	0.882	0.625	0.911	0.882	0.695

External validation set										
	LR	0.781 (0.710, 0.848)	0.651	0.714	0.598	0.887	0.598	0.794	0.887	0.422
	SVM	0.743 (0.664, 0.817)	0.627	0.680	0.543	0.909	0.543	0.853	0.909	0.408
	NB	0.690 (0.605, 0.771)	0.627	0.708	0.620	0.826	0.620	0.647	0.826	0.386
	DT	0.547 (0.451, 0.641)	0.556	0.636	0.533	0.790	0.533	0.618	0.790	0.328
	KNN	0.699 (0.612, 0.782)	0.540	0.592	0.457	0.840	0.457	0.765	0.840	0.342
	AdaBoost	0.548 (0.449, 0.645)	0.548	0.632	0.533	0.778	0.533	0.588	0.778	0.317
	Whole-tumor	0.604 (0.505, 0.705)	0.368	0.455	0.311	0.852	0.311	0.692	0.852	0.150
	Clinical/Metabolic	0.646 (0.544, 0.735)	0.635	0.716	0.630	0.829	0.630	0.647	0.829	0.393
	Spatiotemporal heterogeneity	0.810 (0.740, 0.874)	0.675	0.717	0.571	0.963	0.571	0.943	0.963	0.458

AUC = area under curve, LR = logistic regression, SVM = support vector machine, NB = naive bayes, KNN = k-nearest neighbors, DT = decision tree, AdaBoost = adaptive boosting.

Supplementary Table 4. The performance of spatiotemporal heterogeneity model across subgroups in the validation sets.

Set	Model	AUC (95% CI)	Accuracy	F1 score	Recall	Precision	Sensitivity	Specificity	Positive prediction	Negative prediction
Internal validation set										
	Whole	0.821 (0.746, 0.892)	0.763	0.732	0.625	0.882	0.625	0.911	0.882	0.695
	LUSC	0.831 (0.733, 0.923)	0.806	0.760	0.655	0.905	0.655	0.939	0.905	0.756
	LUAD	0.772 (0.609, 0.917)	0.677	0.688	0.579	0.846	0.579	0.833	0.846	0.556
	Definitive Radiotherapy	0.810 (0.701, 0.910)	0.745	0.731	0.655	0.826	0.655	0.846	0.826	0.688
	Non-radiotherapy	0.825 (0.697, 0.923)	0.789	0.733	0.579	1.000	0.579	1.000	1.000	0.704
External validation set										
	Whole	0.810 (0.740, 0.874)	0.675	0.717	0.571	0.963	0.571	0.943	0.963	0.458
	LUSC	0.806 (0.713, 0.890)	0.656	0.667	0.512	1.000	0.500	1.000	1.000	0.476
	LUAD	0.784 (0.655, 0.895)	0.694	0.759	0.638	0.938	0.638	0.867	0.938	0.433
	Definitive Radiotherapy	0.804 (0.714, 0.886)	0.662	0.696	0.532	1.000	0.532	1.000	1.000	0.450
	Non-radiotherapy	0.813 (0.691, 0.913)	0.690	0.735	0.610	0.926	0.610	0.882	0.926	0.484

AUC = area under curve, LUAD = lung adenocarcinoma, LUSC = lung squamous carcinoma.

Supplementary Table 5. Comparison of metabolic parameters in different habitats.

Set	Metabolic parameters	Habitat subregion				*P* vaule					
		Habitat 1	Habitat 2	Habitat 3	Habitat 4	Habitat 1 vs Habitat 2	Habitat 1 vs Habitat 3	Habitat 1 vs Habitat 4	Habitat 2 vs Habitat 3	Habitat 2 vs Habitat 4	Habitat 3 vs Habitat 4
Train set											
	SUVmax	15.06±0.42	7.89±0.21	7.94±0.21	11.62±0.37	< 0.001	< 0.001	< 0.001	0.898	< 0.001	< 0.001
	SUVmean	10.12±0.27	5.55±0.16	5.86±0.16	9.22±0.25	< 0.001	< 0.001	0.055	0.210	< 0.001	< 0.001
	MTV	29.47±2.64	4.82±0.86	31.08±2.65	2.22±0.65	< 0.001	0.559	< 0.001	< 0.001	0.347	< 0.001
	TLG	316.99±29.28	24.73±4.05	171.94±13.74	22.28±7.00	< 0.001	< 0.001	< 0.001	< 0.001	0.917	< 0.001
Internal validation set											
	SUVmax	15.22±0.61	8.07±0.30	8.10±2.86	11.71±0.51	< 0.001	< 0.001	< 0.001	0.912	< 0.001	< 0.001
	SUVmean	10.36±0.40	5.49±0.22	5.95±0.22	9.35±0.37	< 0.001	< 0.001	0.135	0.207	< 0.001	< 0.001
	MTV	28.01±3.86	2.90±0.37	30.83±4.26	0.68±0.09	< 0.001	0.490	< 0.001	< 0.001	0.568	< 0.001
	TLG	307.62±46.10	17.24±2.58	175.81±22.86	7.76±1.53	< 0.001	< 0.001	< 0.001	< 0.001	0.795	< 0.001
External validation set											
	SUVmax	14.07±0.58	8.01±0.29	8.04±0.29	11.11±0.42	< 0.001	< 0.001	< 0.001	0.911	< 0.001	< 0.001
	SUVmean	9.93±0.37	6.04±0.21	6.28±0.22	9.15±0.33	< 0.001	< 0.001	0.213	0.496	< 0.001	< 0.001
	MTV	18.45±2.29	3.02±0.77	19.44±2.35	1.40±0.57	< 0.001	0.682	< 0.001	< 0.001	0.504	< 0.001
	TLG	209.06±30.01	18.27±4.26	129.47±17.63	13.49±5.92	< 0.001	0.002	< 0.001	< 0.001	0.849	< 0.001

SUV = standardized uptake value, MTV = metabolic tumor volume, TLG = total lesion glycolysis.

Supplementary Table 6. Correlation of spatial characteristics of each habitat with treatment response.

Habitat subregion	Spatial distribution	Odds ratio	95% CI	*P* value
**Voxel count (×10³)**				
Habitat 1	25.968±1.693	0.996	0.991-1.001	0.147
Habitat 2	3.870±0.488	1.017	0.988-1.046	0.249
Habitat 3	27.652±1.746	0.996	0.991-1.001	0.150
Habitat 4	1.654±0.365	1.025	0.982-1.070	0.262
**Volume fraction**				
Habitat 1	0.430 (0.365-0.468)	0.063	0.008-0.483	0.008
Habitat 2	0.057 (0.027-0.137)	9.668	1.071-87.241	0.043
Habitat 3	0.463 (0.392-0.522)	0.822	0.150-4.505	0.821
Habitat 4	0.013 (0.005-0.037)	59.607	0.766-4519.177	0.064

Habitat 1= PEThigh-CThigh habitat, Habitat 2= PETlow-CTlow habitat, Habitat 3= PETlow-CThigh habitat, Habitat 4= PEThigh-CTlow habitat
